# Chemical elicitation as an avenue for discovery of bioactive compounds from fungal endophytes

**DOI:** 10.3389/fchem.2022.1024854

**Published:** 2022-11-23

**Authors:** Madhaiyan Munusamy, Kuan Chieh Ching, Lay Kien Yang, Sharon Crasta, Martin Muthee Gakuubi, Zhao Yan Chee, Mario Wibowo, Chung Yan Leong, Yoganathan Kanagasundaram, Siew Bee Ng

**Affiliations:** ^1^ Singapore Institute of Food and Biotechnology Innovation (SIFBI), Agency for Science, Technology and Research (A*STAR), Singapore, Singapore; ^2^ School of Biological Sciences, Nanyang Technological University, Singapore, Singapore

**Keywords:** *Bartalinia* sp., endophytes, chemical elicitors, pestahivin analogues, mangrove

## Abstract

The present study investigated the molecular phylogeny, antimicrobial and cytotoxic activities of fungal endophytes obtained from the A*STAR Natural Organism Library (NOL) and previously isolated from Sungei Buloh Wetland Reserve, Singapore. Phylogenetic analysis based on ITS2 gene suggests that these isolates belong to 46 morphotypes and are affiliated to 23 different taxa in 17 genera of the *Ascomycota* phylum. *Colletotrichum* was the most dominant fungal genus accounting for 37% of all the isolates, followed by *Diaporthe* (13%), *Phyllosticta* (10.9%) and *Diplodia* (8.7%)*.* Chemical elicitation using 5-azacytidine, a DNA methyltransferase inhibitor and suberoylanilide hydroxamic acid, a histone deacetylase inhibitor resulted in an increase in the number of active strains. Bioassay-guided isolation and structural elucidation yielded pestahivin and two new analogues from *Bartalinia* sp. F9447. Pestahivin and its related analogues did not exhibit antibacterial activity against *Staphylococcus aureus* but displayed strong antifungal activities against *Candida albicans* and *Aspergillus brasiliensis,* with IC_50_ values ranging from 0.46 ± 0.06 to 144 ± 18 µM. Pestahivin and its two analogues furthermore exhibited cytotoxic activity against A549 and MIA PACA-2 cancer cell lines with IC_50_ values in the range of 0.65 ± 0.12 to 42 ± 5.2 µM. The finding from this study reinforces that chemical epigenetic induction is a promising approach for the discovery of bioactive fungal secondary metabolites encoded by cryptic gene clusters.

## 1 Introduction

Fungal endophytes constitute a multifarious group of microorganisms that reside within plant tissues without causing any apparent harm or disease to their host (Petrini et al., 1993; Jia et al., 2016). The interactions between fungal endophytes and their host plants are quite complex and may range from mutualistic to antagonistic relationships (Faeth and Bultman, 2002). The outcome of such interactions varies greatly and are influenced by a myriad of factors such as their growth environment with all its variables, genetic traits of the fungi and the host plant in addition to other biotic factors (Jia et al., 2016). Fungal endophytes have been isolated from all types of plants and plant tissues (Rodriguez et al., 2009). Almost all plants studied thus far have been found to host at least one species of fungal endophyte with many plants harboring up to hundreds of endophytic fungal species (Arnold et al., 2000; Faeth and Fagan, 2002). Many studies especially on tropical plants have revealed a remarkable richness and diversity of fungal endophytes, majority of which are found within the phylum *Ascomycota*. Within the ascomycetes, classes *Sordariomycetes* and *Dothideomycetes* have been found to constitute the highest number of foliar fungal endophytes (Vaz et al., 2012; Vaz et al., 2014).

In recent years, endophytic fungi have increasingly attracted interest as an important source of useful natural products with unique chemical structures and diverse bioactivities (Ratnaweera and de Silva, 2017; Uzma et al., 2019; Gakuubi et al., 2021; Singh et al., 2021). Bioprospecting studies of fungal endophytes sourced from diverse ecosystems including terrestrial, mangrove and marine ecosystems have uncovered fungal strains that are capable of producing secondary metabolites with antimicrobial, cytotoxic/anticancer, antioxidant and immunosuppressive activities (Chi et al., 2019; Manganyi and Ateba, 2020; Adeleke and Babalola, 2021). Despite their potential as prolific producers of bioactive molecules, low yields of target compounds coupled with many biosynthetic gene clusters remaining silent under standard laboratory conditions act as key limiting factors for the exploitation of the full biosynthetic capabilities of fungal endophytes (González-Menéndez et al., 2018). To overcome such challenges, numerous strategies have been devised to enhance the activation of silent biosynthetic gene clusters in fungi (Netzker et al., 2015; Baral et al., 2018; Gakuubi et al., 2021; Kantari et al., 2021). Among these approaches is the use of chemical epigenetic modifiers including histone deacetylases (HDAC) and DNA methyltransferases (DNMT) inhibitors. Growth of fungal endophytes in the presence of different chemical elicitors has been shown to induce the expression of silent gene clusters leading to production of new chemical entities or enhanced biosynthesis of compounds produced in low quantities (Deepika et al., 2016; Dwibedi et al., 2019; González-Menéndez et al., 2019).

Sungei Buloh Wetland Reserve, the explored site in this study, is a Nature Reserve in the Northwest of Singapore, covering an area of about 130 ha and is home to the world’s rarest mangroves (Yang et al., 2011). Mangrove ecosystems are unique habitats characterized by high salinity and organic matter and are known to harbor rich biodiversity (Cadamuro et al., 2021). The diversity of endophytic fungi from plant tissues of mangrove plant taxa has attracted much interest in recent years following the discovery of valuable bioactivities from fungi sourced from this unique ecosystem (Hamzah et al., 2018; Hamzah et al., 2020; Cadamuro et al., 2021; Zhou et al., 2022). The aim of the current study therefore was to assess the molecular phylogeny and bioactive potential of endophytic fungi isolated from Sungei Buloh Wetland Reserve in Singapore. Moreover, two chemical epigenetic modifiers; 5-azacytidine, a DNA methyltransferase inhibitor, and suberoylanilide hydroxamic acid (SAHA), a histone deacetylases inhibitor, were evaluated for their capacity to induce or enhance the biosynthesis of bioactive secondary metabolite from fungal endophytes.

## 2 Materials and methods

### 2.1 Sampling site, isolation and culture conditions

Endophytic fungal strains were originally isolated from different mangrove plants and grass tissues collected from Sungei Buloh Wetland Reserve, Singapore (Table 1). Isolated fungal strains were preliminarily identified using cultural and morphological characteristics and deposited at the Natural Organism Library (NOL) housed at Singapore Institute of Food and Biotechnology Innovation (SIFBI) A*STAR, Singapore (Ng et al., 2018). The selected fungal strains were retrieved from -80°C and revived by growth on malt extract agar (MEA, Oxoid, United Kingdom) or potato dextrose agar (PDA, Sigma, United States) for 4–7 days at 24°C.

**TABLE 1:** List of endophytic fungi (*Phylum: Ascomycetes*) isolated from different terrestrial plant tissues and taxonomic identification based on ITS2 gene sequence.

NOL accession	Substrate*	Tissue	Close relatives of ITS2 region of type fungi and reference material in NCBI		Order, family	Classification
			Scientific name	% Similarity		
F6342	*Acanthus* species (Sea holly/Mangrove holly)	Leaf	*Colletotrichum cobbittiense* (NR_163538.1)	100	*Glomerellales; Glomerellaceae*	*Colletotrichum* sp.
F6343	*Acanthus* species (Sea holly/Mangrove holly)	Leaf	*C. cobbittiense* (NR_163538.1)	100	*Glomerellales; Glomerellaceae*	*Colletotrichum* sp.
F6344	*Hevitiera littoris* (mangrove dungun)	Leaf	*C. cobbittiense* (NR_163538.1)	100	*Glomerellales; Glomerellaceae*	*Colletotrichum* sp.
F6345	*Acanthus* species (Sea holly/Mangrove holly)	Leaf	*C. cobbittiense* (NR_163538.1)	100	*Glomerellales; Glomerellaceae*	*Colletotrichum* sp.
F6346	*Hevitiera littoris* (mangrove dungun)	Leaf	*Diaporthe australiana* (NR_168239.1)	**97.3**	*Diaporthales; Diaporthaceae*	*Diaporthe* sp.
F6348	*Hevitiera littoris* (mangrove dungun)	Leaf	*Diplodia cajani* (NR_163672.1)	100	*Botryosphaeriales; Botryosphaeriaceae*	*Diplodia* sp.
F6351	*Avicennia rumphianni*/*Acrostictum* (*Pteridaceace*)	Stem/Leaf	*Neodevriesia hilliana* (NR_145098.1)	**93.5**	*Mycosphaerellales; Neodevriesiaceae*	*Mycosphaerellales*
F6355	*Eupharbaceace*	Leaf	*C. cobbittiense* (NR_163538.1)	99.7	*Glomerellales; Glomerellaceae*	*Colletotrichum* sp.
F6357	*Dillenia suffroticose*	Leaf	*Phyllosticta fallopiae* (NR_147316.1)	100	*Botryosphaeriales; Phyllostictaceae*	*Phyllosticta* sp.
F6369	*Acanthus* species (Sea holly/Mangrove holly)	Stem	*C. cobbittiense* (NR_163538.1)	99.7	*Glomerellales; Glomerellaceae*	*Colletotrichum* sp.
F6371	*Hevitiera littoris* (mangrove dungun)	Leaf	*C. cobbittiense* (NR_163538.1)	100	*Glomerellales; Glomerellaceae*	*Colletotrichum* sp.
F6373	*Hevitiera littoris* (mangrove dungun)	Stem	*D. cajani* (NR_163672.1)	100	*Botryosphaeriales; Botryosphaeriaceae*	*Diplodia* sp.
F6376	*Calophyllum inophyllum* (mangrove)	Leaf	*P. fallopiae* (NR_147316.1)	100	*Botryosphaeriales; Phyllostictaceae*	*Phyllosticta* sp.
F6378	*Calophyllum inophyllum* (mangrove)	Leaf	*P. fallopiae* (NR_147316.1)	99.7	*Botryosphaeriales; Phyllostictaceae*	*Phyllosticta* sp.
F6379	*Avicennia rumphianni* (mangrove-Api-api bulu)	Leaf	*Diaporthe searlei* (NR_168241.1)	**97.9**	*Diaporthales; Diaporthaceae*	*Diaporthe* sp.
F6381	*Avicennia rumphianni*/*Acrostictum* (*Pteridaceace*)	Stem/Leaf	*C. cobbittiense* (NR_163538.1)	100	*Glomerellales; Glomerellaceae*	*Colletotrichum* sp.
F6383	*Excoecaria* (*Eupharbaceace*)	Leaf	*C. cobbittiense* (NR_163538.1)	99.7	*Glomerellales; Glomerellaceae*	*Colletotrichum* sp.
F6384	*Excoecaria* (*Eupharbaceace*)	Leaf	*C. cobbittiense* (NR_163538.1)	99.7	*Glomerellales; Glomerellaceae*	*Colletotrichum* sp.
F6385	*Excoecaria* (*Eupharbaceace*)	Leaf	*C. cobbittiense* (NR_163538.1)	99.7	*Glomerellales; Glomerellaceae*	*Colletotrichum* sp.
F6386	*Excoecaria* (*Eupharbaceace*)	Stem	*C. cobbittiense* (NR_163538.1)	99.7	*Glomerellales; Glomerellaceae*	*Colletotrichum* sp.
F6387	*Excoecaria* (*Eupharbaceace*)	Stem	*D. cajani* (NR_163672.1)	100	*Botryosphaeriales; Botryosphaeriaceae*	*Diplodia* sp.
F6388	*Excoecaria* (*Eupharbaceace*)	Stem	*D. australiana* (NR_168239.1)	**96.9**	*Diaporthales; Diaporthaceae*	*Diaporthe* sp.
F6389	*Dillenia suffroticose* (mangrove)	Leaf	*D. searlei* (NR_168241.1)	**97.9**	*Diaporthales; Diaporthaceae*	*Diaporthe* sp.
F6390	*Dillenia suffroticose* (mangrove)	Leaf	*C. cobbittiense* (NR_163538.1)	100	*Glomerellales; Glomerellaceae*	*Colletotrichum* sp.
F6394	*Dillenia suffroticose* (mangrove)	Stem	*C. cobbittiense* (NR_163538.1)	100	*Glomerellales; Glomerellaceae*	*Colletotrichum* sp.
F6406	*Dillenia suffroticose* (mangrove)	Stem	*Trichoderma breve* (NR_154574.1)	99.7	*Hypocreales; Hypocreaceae*	*Trichoderma* sp.
F6417	*Acanthus* species (Sea holly/Mangrove holly)	Stem	*Stagonosporopsis lupini* (NR_160205.1)	98.9	*Pleosporales; Didymellaceae*	*Stagonosporopsis* sp.
F6419	*Avicennia rumphianni* (mangrove- Api-api bulu)	Leaf	*D. searlei* (NR_168241.1)	**97.9**	*Diaporthales; Diaporthaceae*	*Diaporthe* sp.
F6420	*Avicennia rumphianni*/*Acrostictum* (*Pteridaceace*)	Stem/Leaf	*C. cobbittiense* (NR_163538.1)	99.7	*Glomerellales; Glomerellaceae*	*Colletotrichum* sp.
F6422	*Avicennia rumphianni*/*Acrostictum* (*Pteridaceace*)	Stem/Leaf	*D. searlei* (NR_168241.1)	**97.9**	*Diaporthales; Diaporthaceae*	*Diaporthe* sp.
F6423	*Acrostictum* (*Pteridaceace*)	Stem	*P. fallopiae* (NR_147316.1)	100	*Botryosphaeriales; Phyllostictaceae*	*Phyllosticta* sp.
F6424	*Nephrolepideceace*	Leaf	*C. cobbittiense* (NR_163538.1)	99.6	*Glomerellales; Glomerellaceae*	*Colletotrichum* sp.
F6426	*Eupharbaceace*	Stem	*D. cajani* (NR_163672.1)	100	*Botryosphaeriales; Botryosphaeriaceae*	*Diplodia* sp.
F6427	*Rhizophora* (mangrove)	Stem	*Leptosillia wienkampii* (NR_164067.1)	**90.6**	*Xylariales; Leptosilliaceae*	*Xylariales*
F6428	*Rhizophora* (mangrove)	Stem	*Phyllosticta schimicola* (NR_147356.1)	**95.3**	*Botryosphaeriales; Phyllostictaceae*	*Botryosphaeriales*
F6430	*Dillenia suffroticose* (mangrove)	Leaf	*Aspergillus fumigatus* (NR_121481.1)	99.7	*Eurotiales; Aspergillaceae*	*Aspergillus* sp.
F6432	*Dillenia suffroticose* (mangrove)	Stem	*C. cobbittiense* (NR_163538.1)	100	*Glomerellales; Glomerellaceae*	*Colletotrichum* sp.
F6507	*Calophyllum inophyllum* (mangrove)	Leaf	*Zasmidium podocarpi* (NR_156004.1)	**94.0**	*Mycosphaerellales; Mycosphaerellaceae*	*Mycosphaerellales*
F6509	*Rhizophora* (mangrove)	Stem	*Massaria campestris* (NR_137583.1)	**81.9**	*Pleosporales; Massariaceae*	*Pleosporales*
F6529	*Calophyllum inophyllum* (mangrove)	Leaf	*Corynespora torulosa* (NR_145181.1)	**98.0**	*Pleosporales; Corynesporascaceae*	*Corynespora* sp.
F9446	Grass	Flower	*Preussia polymorpha* (NR_137729.1)	**88.9**	*Pleosporales; Sporormiaceae*	*Pleosporales*
F9447	Grass	Flower	*Bartalinia pondoensis* (NR_153599.1)	100	*Xylariales; Sporocadaceae*	*Bartalinia* sp.
F9448	*Acanthus* species (Sea holly/Mangrove holly)	Leaf	*Neopyrenochaeta annellidica* (NR_170042.1)	99.0	*Pleosporales; Neopyrenochaetaceae*	*Neopyrenochaeta* sp.
F9449	Grass	Flower	*Robillarda terrae* (NR_132902.1)	99.3	*Xylariales; Sporocadaceae*	*Robillarda* sp.
F9452	Grass	Flower	*Fusarium hainanense* (NR_164597.1)	100	*Hypocreales; Nectriaceae*	*Fusarium* sp.
F9456	Grass	Flower	*P. polymorpha* (NR_137729.1)	**88.9**	*Pleosporales; Sporormiaceae*	*Pleosporales*

*Sample collection from Terrestrial plant species including mangrove species and grass flower, Sungei Buloh, Singapore.

**NCBI Blast search hits based on ITS2 gene sequence from fungi type and reference material.

Values in bold indicates sequence similarities ≤97% when compared with reference species from the GenBank database.

### 2.2 DNA extraction, sequencing and phylogenetic analysis

The mycelia of the fungal samples were harvested and subjected to cryogenic grinding with liquid nitrogen before DNA extraction using the DNeasy PowerSoil Pro Kit (Qiagen, Germany) following the manufacturer’s instructions. PCR amplification of the ITS2 region was performed using primer pair ITS4/ITS86F (Vancov and Keen, 2009). The final PCR reactions contained 12.8 µL of nuclease free water, 2 µL of 10 × DreamTaq Green Buffer (ThermoFisher Scientific), 2 µL of 2 mM dNTP Mix (1st BASE, Axil Scientific. Singapore), 0.2 µL of DreamTaq DNA Polymerase (ThermoFisher Scientific) and 1 µL of each primer (10 µM) in their respective pairs. PCR was performed using Veriti^TM^ Thermal Cycler (Applied Biosystems, United States) using an initial denaturation step at 95°C for 5 min, followed by 40 cycles at 95°C for 30 sec, 60°C for 30 sec, 72°C for 30 sec, with a final extension step of 10 min of 72°C. Successful amplification of target region was confirmed by visualizing 1 µL of the products following Gel electrophoresis and the products were purified using the MEGAquick-spin™ Total Fragment DNA Purification Kit (Intron Biotechnology, Republic of Korea) before sending the amplicons to 1^st^ BASE (Axil Scientific, Singapore) for DNA sequencing. Using the software Benchling (https://benchling.com), the consensus sequences were constructed by alignment of the forward and reverse sequences. Sequence regions upstream of the forward primer and downstream of the reverse primer were removed from the aligned sequences and the resultant sequences uploaded into BLASTn (Altschul et al., 1990) for strain identification. Sequence alignment and phylogenetic trees were generated using MEGA 7 software (Kumar et al., 2016). All primers used for phylogenetic analyses in this study are listed in Supplementary Table S1. For multilocus sequence analysis (MLSA), gene sequences of large subunit ribosomal ribonucleic acid (LSU), small subunit ribosomal ribonucleic acid (SSU), internal transcribed spacer 2 (ITS2), translation elongation factor 1-alpha (*TEF-1α*), RNA polymerase II second largest subunit (RPB2) and *β-*tubulin gene sequence (TUB) were obtained from F9447 genome data and those of closest relatives retrieved from NCBI database and recent references (Phookamsak et al., 2019; Tibpromma et al., 2020). DNA gene sequences were aligned using the ClustalW algorithm in MEGA 7. ITS sequences were deposited in NCBI GenBank with accession numbers OP001746- OP001791 (Supplementary Table S2). The sequences for the nrLSU, ITS, RPB2, *TEF1-α*, and beta-tubulin, for this strain are available in the GenBank database and their accession numbers are OP002023, OP001787, OP828688, OP828689, and OP828690 respectively (Supplementary Table S4). For each gene, sequences were aligned individually and then concatenated in a super-gene alignment used for construction of a phylogenetic tree in MEGA 7 using Maximum Likelihood method with bootstrap values set at 500 replications (Kumar et al., 2016).

### 2.3 Small and large-scale fermentation and extraction of fungal crude extracts

The endophytic isolates were subjected to small-scale fermentation for extract generation by inoculating 3 mm fungal mycelial disc of 4–7 days old fungal culture into 10 ml pre-sterilized CF02LB and CF18LB media (Gakuubi et al., 2022) in 50 ml Erlenmeyer flasks. The flasks were then incubated at 24°C, 200 rpm for 14 days. To study the effect of chemical epigenetic modifiers, 3 mm fungal mycelial disc (3 disc/flask) were added into 20 ml of CF02LB and CF18LB production media separately supplemented with DMSO-dissolved 5-azacytidine and SAHA, resulting in the final concentrations of 50 µM 5-azacytidine and 100 µM SAHA. Equal amounts of DMSO were added to the control groups. The cultures were fermented in shaker flasks at 24°C and 200 rpm for 14 days. After 14 days of fermentation, cultures were freeze-dried and extracted with equal volume of methanol (VWR Chemicals #20864) by overnight shaking (200 rpm, 24°C), followed by filtration through filter paper (Whatman No. 4; 20–25 μM pore size) and filtrates were dried using a centrifugal concentrator (MiVac Quattro, Thermo Fisher Scientific). The methanol extracts were subjected to biological testing followed by chemical analysis of active samples.

### 2.4 Antimicrobial and cytotoxic activity of fungal secondary metabolites

Three microbial pathogens including bacterium *Staphylococcus aureus* strain ATCC 25923, a yeast *Candida albicans* strain ATCC 10231 and a mould *Aspergillus brasiliensis* strain ATCC 16404 were used to assess the antimicrobial activity of fungal secondary metabolites. Primary antimicrobial screening of fungal extracts was performed at concentration of 200 μg/ml in duplicate using the 384-well assay described previously (Gakuubi et al., 2022). *Aspergillus brasiliensis* screen was similarly done as described previously for mould pathogens with the exception that, 2.5 × 10^3^ spores/mL were seeded. Gentamicin and amphotericin B were used as the standard control in bacterial and fungal screens with starting final assay concentrations of 25 and 20 μg/mL, respectively. To evaluate the cytotoxic activities of endophytic fungal extracts, all the crude extracts were tested against cancer cell lines A549, PANC-1 and MIA PaCa-2 following the method described previously (Gakuubi et al., 2022). For both the antimicrobial and cytotoxicity assays, crude extracts that revealed promising biological activity (average % inhibition ≥ 50) following primary screening were subjected to dose-response testing for confirmation of activity with a starting concentration of 200 μg/ml in an eight-point, two-fold serial dilution microplate assay. Dose-response testing for isolated compounds was carried out in triplicate in a twelve-point, two-fold serial dilution assay with a starting concentration of 100 μg/ml.

### 2.5 Large-scale fermentation, dereplication, compound isolation and structure elucidation

Active extracts were analyzed according to a dereplication procedure as described in the literature (Butler et al., 2012). Strain F9447 which was found to contain active constituents of interest, was fermented in large-scale to obtain sufficient crude extracts for compound isolation and structure elucidation. Firstly, the strain was sub-cultured on MEA for 7 days at 24°C. Three agar plugs of 5 mm in diameter with fungal mycelia were inoculated into each of the 60 × 250 mL Erlenmeyer flasks containing 50 ml of either CF02LB or CF18LB fermentation media. The culture media were supplemented with 50 µM 5-azacytidine except for the control flasks. The cultures were incubated at 24°C with shaking at 200 rpm for 14 days. At the end of the incubation period, the cultures were harvested and freeze-dried for 3–5 days before extraction overnight with methanol. Solid materials were then removed from the extraction mixture *via* filtration using Whatman^TM^ Grade 4 filter paper (GE Healthcare Life Sciences) before the filtrate was dried using a rotary evaporator.

The dried extract was combined and partitioned between aqueous methanol (MeOH:H_2_O in a ratio of 1:1, 800 mL) and CH_2_Cl_2_ (400 mL), followed by extracting the aqueous methanol phase with CH_2_Cl_2_ (2 × 400 mL) (Kupchan et al., 1973). The organic layer was combined and evaporated to dryness using rotary evaporation. The dried CH_2_Cl_2_ crude extract (13.8 g) was redissolved in CH_2_Cl_2_:MeOH (1:1) and separated by size exclusion chromatography on Sephadex LH-20 (CH_2_Cl_2_:MeOH = 1:1) and the sample (1.5 g) was subjected to silica gel column chromatography eluted using 1–5% MeOH/CH_2_Cl_2_ to obtain an enriched fraction containing pestahivin analogues (122.3 mg). The mixture of pestahivin analogues was further purified by C_18_ reversed-phase preparative HPLC (solvent A: H_2_O + 0.1% HCOOH, solvent B: acetonitrile + 0.1% HCOOH; flow rate: 30 mL/min, gradient conditions: 50:50 isocratic for 5 min; followed by 50%–65% of solvent B over 15 min, 65%–100% of solvent B over 42 min, and finally isocratic at 100% of solvent B for 10 min) to give 37 mg of pestahivin (**1**) (RT 45 min), 20.5 mg of pestahivin B (**2**) (RT 34 min) and 7.3 mg of pestahivin C (**3**) (RT 39 min). The structure of pestahivin was confirmed by comparison of accurate mass from HRMS data and 1D/2D nuclear magnetic resonance (NMR) data with the literature values (Itazaki et al., 1995).

### 2.6 General analytical chemistry procedures

Optical rotations were recorded using JASCO P-2000 digital polarimeter. NMR spectra were acquired on Bruker DRX-400 NMR spectrometer equipped with Cryoprobe. Preparative HPLC analysis were performed with Agilent 1,260 Infinity Preparative-Scale LC/MS Purification System and Agilent 6130B single quadrupole mass spectrometer using Agilent 5 Prep C18 column (10 × 30 mm). UV absorptions were measured, and HPLC-MS was done using Agilent UHPLC 1290 Infinity hyphenated with a diode array detector (DAD). HRMS spectra were recorded in positive ionization mode on an Agilent 6,540 accurate-mass quadrupole time-of-flight (QTOF) mass spectrometer equipped an electrospray ionization (ESI) source. For over 8.6 min, a gradient condition of 98% water (0.1% formic acid) to 100% acetonitrile (0.1% formic acid) was employed, using an Acquity UPLC BEH C18 (2.1 × 50 mm, 1.7 µm) column, at flow rate of 0.5 ml/min. Collision energy of 40 eV was applied in the acquisition of MS^2^ spectra. The operating parameters for QTOF were the same as in previously reported (Sirota et al., 2018). The acquired MS^2^ data were converted to mzXML format using MSConvert for molecular networking analysis. Molecular networking was performed using GNPS (gnps.ucsd.edu) workflow using spectral clustering algorithm with a minimum matched fragment of 6 and a minimum pairs cosine of 0.7. The resulting molecular network was visualized and processed using Cytoscape version 3.9.0.

## 3 Results and discussion

### 3.1 Isolation, identification, and diversity of culturable fungal endophytes

Fungal endophytes were previously isolated from different mangrove plant samples collected from Sungei Buloh Wetland Reserve area, Singapore. The stocks were maintained at -80°C in A*STAR Natural Organism Library (NOL) housed at the Singapore Institute of Food and Biotechnology Innovation (SIFBI). A total of 46 isolates were successfully revived. In order to verify the taxonomic placement of the fungal isolates, gDNA was extracted and amplified *via* PCR sequencing of the ITS2 region and LSU region for the accurate identification of species/genus level (Vancov and Keen, 2009; Raja et al., 2017; Chi et al., 2019). The Blastn search for the ITS2 and LSU sequences and phylogeny analysis revealed that all isolates fall within the phylum *Ascomycota*. The fungal strains were categorized into 46 morphotypes based on cultural characteristics and were affiliated to 19 morphological species within 17 genera and 8 orders (Supplementary Figure S1). Many phylogenetic studies have shown that the majority of fungal endophytes belong to the phylum *Ascomycota* with a few representatives from other *phyla* such *Basidiomycota* and *Mucoromycota* (Crozier et al., 2006; He et al., 2012; Koukol et al., 2012), and the same finding are reflected in this study. The most abundant orders included *Glomerellales* (37%) and *Botryosphaeriales* (20%) followed by *Diaporthales* (13%). At the genus level, *Colletotrichum* was the most dominant grouping of fungi*. Colletotrichum* is an important plant pathogen but also occurs in other lifestyles such as endophytes, saprobes and rarely entomopathogens (Dean et al., 2012; da Silva et al., 2020; Yan et al., 2019; Peters et al., 2020). The genus comprises of more than 700 species including several mangroves species (Chaeprasert et al., 2010; De Souza Sebastianes et al., 2013; Rabha et al., 2016; Nurunnabi et al., 2020; Luo et al., 2021). A previous study cited *Colletotrichum* as the most dominant genus of endophytic fungi isolated from four different habitats of Singapore (Gakuubi et al., 2022). Besides *Colletotrichum*, other fungal genera that have been isolated in abundance among the mangrove plants include *Aspergillus, Cladosporium, Diaporthe, Dothiorella, Emericella, Glomerella, Lasiodiplodia, Leptosphaerulina, Nodulisporium, Penicillium, Pestalotiopsis, Phoma, Phomopsis, Phyllosticta, Pleosporales, Trichoderma,* and *Xylaria* (de Souza Sebastianes et al., 2013; Cadamuro et al., 2021). Our findings also revealed that members belonging to Genus *Diaporthe, Diplodia, Phyllosticta*, and *Preussia* were found in higher frequency while *Aspergillus, Bartalinia, Corynespora*, *Fusarium, Leptosillia, Neodevriesia, Neopyrenochaeta, Robillarda, Stagonosporopsis, Trichoderma* and *Zasmidium* occurred at a lower frequency (Table 1). ITS/28S phylogentic analysis revealed that several isolates have distinct lineage (from 95–98% similarity) and seven isolates (F6351, F6427, F6428, F6507, F6509, F9446, and F9456) had sequence similarities ≤95% when compared to the reference species from the GenBank database and thus were not assignable at the genera level (Table 1 and Supplementary Figure S1). The detailed information of all the 46 strains, including their host plant species, closest relatives based on ITS2 region sequencing, and taxonomy details are summarized in Table 1.

### 3.2 Bioactivity of extracts derived from fungal endophytes

Chemical elicitation using small molecules to perturb the chromatin machinery has been identified as an important tool for the activation of cryptic secondary metabolite pathways in fungi (Triastuti et al., 2019; Pillay et al., 2022). In the current study, fungal endophytes were explored for their ability to produce metabolites with antibacterial, antifungal, and cytotoxic activities. For this purpose, all 23 fungal isolates selected following phylogenetic analysis were grown in two liquid media; CF02LB and CF18LB with and without the addition of two chemical elicitors. The resultant 138 crude extracts were tested for growth inhibitory effect against three microbial pathogens (*S. aureus, C. albicans* and *A. brasiliensis*) and three cancer cell lines (A549, MIA PaCa-2 and PANC-1). Supplementary Figures S2, S3 show the antimicrobial and cytotoxic activity distribution of the extracts generated from 23 fungal strains grown under different fermentation regimes**.**

**FIGURE 1:**
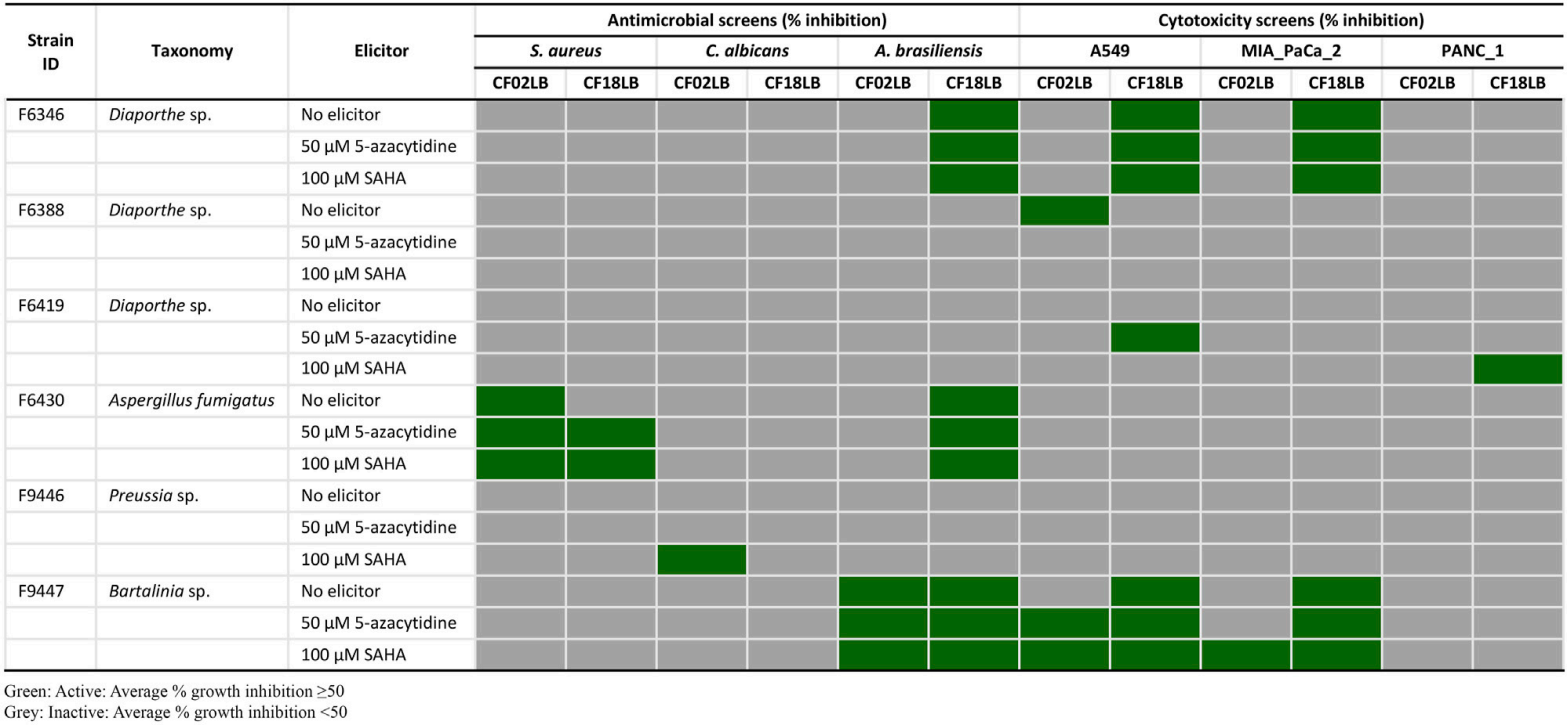
Antimicrobial and cytotoxic activity profiles of six bioactive fungal strains grown in CF02LB and CF18LB media in the presence and absence of chemical elicitors.

**FIGURE 2:**
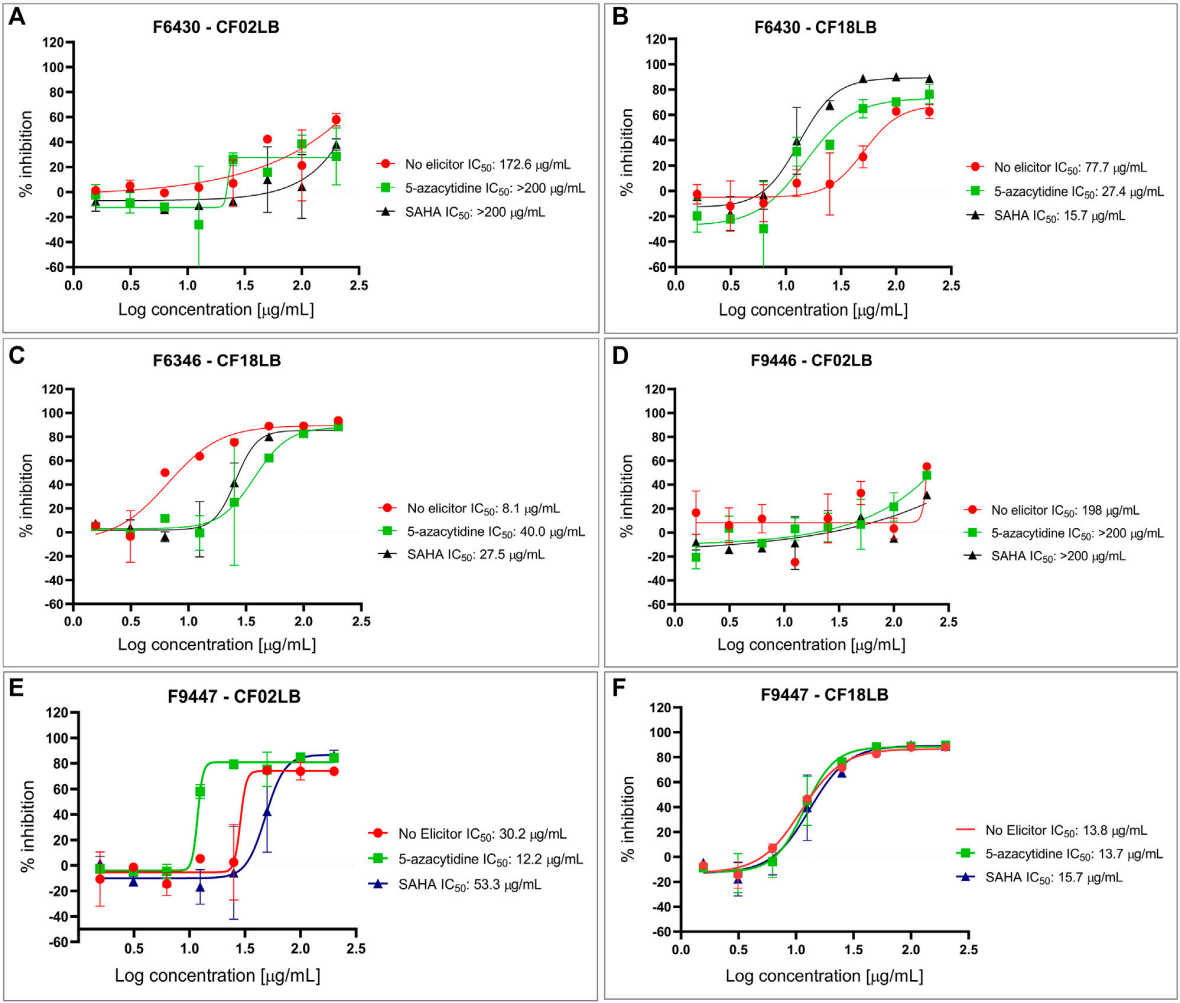
Dose response testing of extracts from F6430 strain grown in CF02LB **(A)** and CF18LB **(B)**, F6346 strain grown in CF18LB **(C)**, F9446 strain grown in CF02LB **(D)** and F9447 strain grown in CF02LB **(E)** and CF18LB **(F)** against *Aspergillus brasiliensis*.

From the 23 isolates studied, 6 strains (representing 26% of all the studied strains) exhibited antimicrobial and/or cytotoxic activity against at least one of the tested microbial pathogens and cancer cell lines. Figure 1 summarizes the bioactivity profiles of these 6 strains. Among the 138 tested extracts, 5 (3.6%), 1 (0.7%), and 12 (8.7%) revealed inhibitory activity against *S. aureus, C. albicans* and *A. brasiliensis*, respectively. Out of the 16 extracts showing antimicrobial activity, 9 (56%) and 7 (44%) were derived from fungal strains grown in CF18LB and CF02LB media, respectively. Regarding cytotoxic activity, 10 (7.3%), 7 (5.1%), and 1 (0.7%) revealed cytotoxic activity against A549, MIA PaCa-2 and PANC-1 cell lines, respectively, with 8 (73%) and 3 (27%) of the 11 active extracts arising from fungal strains grown in CF18LB and CF02LB media, respectively. Differences in the bioactivity of fungal extracts derived from the same strains when grown in different media have been reported in the literature (Gakuubi et al., 2022). Extracts from four fungal strains; F6430, F9447, F6346, and F9446 revealed promising antimicrobial activity. Of these, extracts from F6430 and F9447 grown in both CF02LB and CF18LB media exhibited antimicrobial activity while for F6346 and F9446, antimicrobial activity was observed from extracts derived from CF18LB and CF02LB media respectively (Figure 1). Thus, these six extracts were subjected to dose-response testing for confirmation of activity. The results for dose-response testing for antifungal activity of the aforementioned six extracts against *Aspergillus brasiliensis* are shown in Figure 2. For the extracts derived from F9447 grown in CF02LB under three different regimes, samples prepared from fermentation of the fungus in the presence of 5-azacytidine presented the best antifungal activity against *A. brasiliensis* with an IC_50_ value of 12 μg/mL compared with extracts derived from the fungus grown in the absence of elicitor and in the presence of SAHA, whose IC_50_ values were 30 and 53 μg/mL, respectively (Figure 2).

### 3.3 Chemical dereplication of active extracts

Chemical dereplication was performed on extracts obtained from six fungal strains cultivated under various conditions, which exhibited antimicrobial and cytotoxic activities (Figure 1). The extracts were subjected to HRMS analysis for compounds identification by matching their accurate mass against Dictionary of Natural Products (DNP) database for fungal secondary metabolites (http://dnp.chemnetbase.com). This resulted in the putative identification of three known bioactive molecules from 2 strains (Supplementary Table S3). Among these, deacetoxyfumigaclavine C (*m/z* 309.2298) and fumitremorgin C (*m/z* 380.1967) identified in F6430 fermented in CF18LB media in the presence of 50 µM 5-azacytidine or 100 µM SAHA demonstrated antifungal activities while new pestahivin analogues (*m/z* 935.5953; *m/z* 949.6113) and pestahivin (*m/z* 977.6425) identified in F9447 fermented in CF02LB in the presence of 50 µM 5-azacytidine or 100 µM SAHA presented both antifungal and cytotoxic activities. Unknown antibacterial compounds (*m/z* 939.4487 and *m/z* 1,009.5256) were identified in F6430 fermented in CF18LB media in the presence of 100 µM SAHA (Supplementary Table S3). As the observed antibacterial activity is weak, strain F6430 was not pursued further.

**FIGURE 3:**
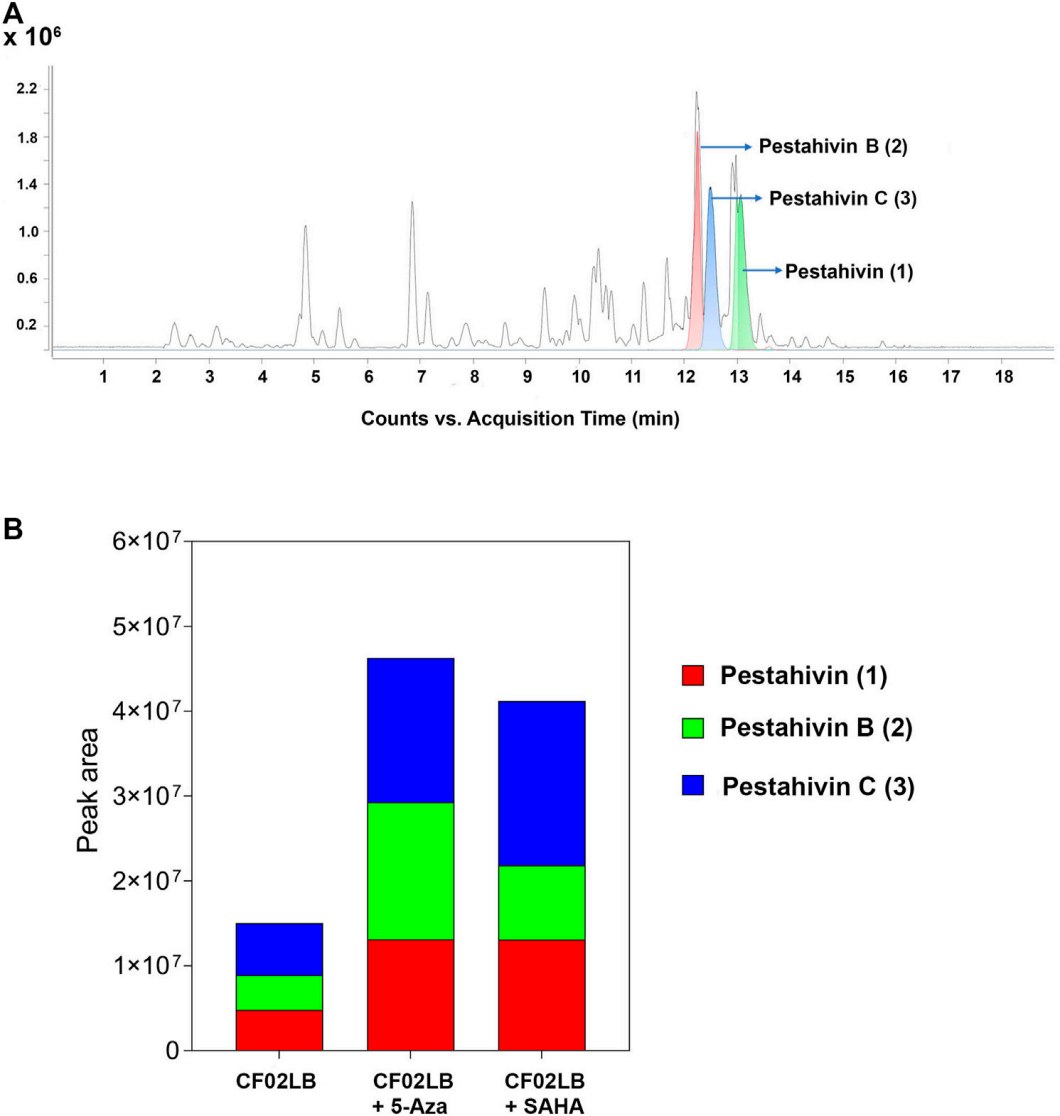
Enhancement of the biosynthesis of pestahivin and its analogues by chemical elicitation in *Bartalinia* sp. F9447. **(A)** LC-MS chromatograms obtained from small-scale fermented extracts from *Bartalinia* sp. F9447 grown in the presence of 50 µM 5-azacytidine, EIC of compounds **1**–**3** are shown in green, red, and blue, respectively. **(B)** Comparison of relative abundance (peak area) of various pestahivin analogues from F9447 cultures grown with or without the chemical elicitors.

Based on the chemical dereplication studies, the major compounds pestahivin and its related new analogues were suspected to account for the antifungal and cytotoxic activities observed in F9447. In addition, in the dose-response study the antifungal activity of F9447 grown in CF02LB was enhanced in the presence of 5-azacytidine (Figure 2E). Hence, the effects of this chemical elicitor on the production of these new pestahivin analogues were investigated. Addition of 50 µM of 5-azacytidine was found to enhance the production of pestahivin (**1**) (*m/z* 977.6425) in F9447 by ∼3 -fold, and pestahivin B (**2**) (*m/z* 935.5953) and pestahivin C (**3**) (*m/z* 949.6113) by 3-4-fold in F9447 (Figures 3A,B). On the basis of chemical dereplication studies, active constituents of interest were identified from strain F9447 grown in CF02LB media in the presence of 5-azacytidine. Therefore, this strain was progressed to large scale fermentation under the same growth conditions for compound isolation and structure elucidation.

### 3.4 Isolation of pestahivin (1), pestahivin B (2) and pestahivin C (3)

**FIGURE 4:**
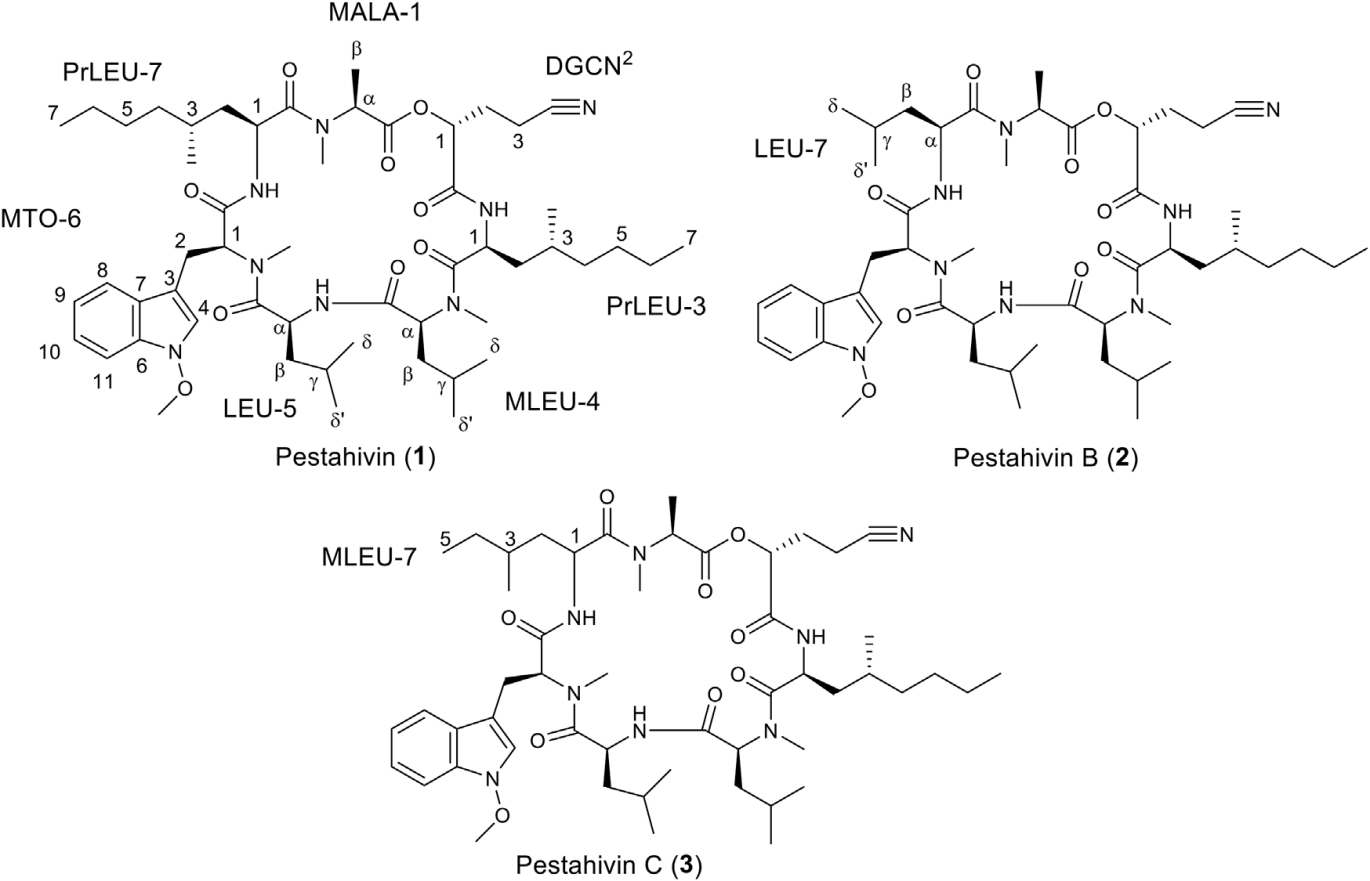
Structures of pestahivin (**1**), pestahivin B (**2**) and C (**3**).

Fungal strain F9447 was fermented in the presence of 50 µM of 5-azacytidine. The MeOH extract of F9447 was obtained to isolate pestahivin (**1**) and the two new pestahivin analogues, pestahivin B (**2**) and pestahivin C (**3**) that were responsible for the observed antimicrobial activities (Figure 4).

Compound **1** was isolated as white amorphous powders with ${\left[\alpha \right]}_{D}^{23}$ − 41 (MeOH). HR-ESI-MS showed an [M+H]^+^ peak at *m/z* 977.6439 and established the molecular formula C_53_H_84_N_8_O_9_. The presence of 5 aromatic signals at δ_H_ 7.07, 7.20, 7.36, 7.43 and 7.63 in the ^1^H NMR spectrum indicated an indole moiety (Table 2 and Supplementary Figure S7). The NMR data (Table 2) exhibited features of a peptide-derived compound supported by the presence of 3 exchangeable -NH proton signals (δ_H_ 7.85, 8.33, 8.60) and 7 amide/carbonyl carbon signals (δ_C_ 167.3, 167.9, 169.8, 170.0, 170.4, 171.5, and 172.6). In addition, the ^1^H/^13^C NMR, HRMS, and specific rotation data of compound **1** were consistent with the literature values (Itazaki et al., 1995). Furthermore, the absolute configuration of **1** has been previously established *via* X-ray analysis, which showed that all amino acids in **1** had L-configuration (Hommel et al., 1996). Based on spectroscopic data comparison, compound **1** was identified as the known cyclopeptolide, pestahivin (HUN-7293) (Itazaki et al., 1995; Boger et al., 1999).

**TABLE 2:** NMR spectral data^*a*^ of pestahivin (**1**).

Amino acid residue	Position	^13^C	^1^H, mult. (*J* = Hz)
N-Me-Ala (MALA-1)	N-Me	36.7, CH_3_	3.19, s
	α	58.3, CH	3.98, q (6.8)
	β	13.4, CH_3_	1.36, d (6.7)
	CO	170.0, C	
(*R*)-2-hydroxy-4-cyanobutyric acid (DGCN-2)	1	73.6, CH	4.88, dd (3.5, 10.4)
	2	26.4, CH_2_	1.71, m; 2.00, m
	3	13.1, CH_2_	2.42, m; 2.46, m
	4	120.7, C	
	CO	167.3, C	
(4*R*)-5-propyl-L-leucine (PrLEU-3)	NH		7.85, d (9.6)
	1	46.5, CH	4.68, q (7.7)
	2	39.4, CH_2_	1.50, m; 1.50, m
	3	28.1, CH	1.27, m
	3-Me	20.0, CH_3_	0.80, d (6.4)
	4	36.3, CH_2_	1.07, m; 1.18, m
	5	28.5, CH_2_	1.17, m; 1.27, m
	6	22.4, CH_2_	1.24, m; 1.24, m
	7	13.9, CH_3_	0.86, t (6.9)
	CO	169.8, C	
N-Me-Leu (MLEU-4)	N-Me	27.9, CH_3_	2.40, s
	α	57.3, CH	4.09, m
	β	35.9, CH_2_	1.57, m; 1.64, m
	γ	24.4, CH	1.35, m
	δ	21.6, CH_3_	0.94, d (6.7)
	δ’	23.3, CH_3_	0.92, d (6.9)
	CO	171.5, C	
Leu (LEU-5)	N-H		8.33, d (6.4)
	α	47.4, CH	4.09, m
	β	36.5, CH_2_	−0.70, m; 1.36, m
	γ	22.8, CH	1.23, m
	δ	18.1, CH_3_	−0.20, d (6.6)
	δ'	22.5, CH_3_	0.40, d (6.4)
	CO	172.6, C	
*N*’-methoxy-*N*-methyl-L-tryptophan (MTO-6)	N-Me	28.6, CH_3_	2.78, s
	1	60.4, CH	4.83, dd (4.1, 10.7)
	2	23.1, CH_2_	3.05, m; 3.10, m
	3	106.2, C	
	4	122.9, CH	7.36, s
	5-OMe	65.8, CH_3_	4.01, s
	6	131.7, C	
	7	123.4, C	
	8	118.6, CH	7.63, d (7.7)
	9	119.7, CH	7.07, t (7.0)
	10	122.3, CH	7.20, t (7.7)
	11	108.3, CH	7.43, d (8.4)
	CO	167.9, C	
(4*R*)-5-propyl-L-leucine (PrLEU-7)	N-H		8.60, d (9.9)
	1	46.1, CH	4.91, m
	2	38.4, CH_2_	1.26, m; 1.51, m
	3	28.2, CH	0.87, m
	3-Me	18.7, CH_3_	0.87, d (6.1)
	4	36.5, CH_2_	1.05, m; 1.30, m
	5	28.7, CH_2_	1.16, m; 1.28, m
	6	22.3, CH_2_	1.24, m; 1.24, m
	7	14.0, CH_3_	0.86, t (6.9)
	CO	170.4, C	

^*a*1^H (400 MHz) and

^13^C (100 MHz) in DMSO-*d*_*6*_. Assignments based on COSY, HSQC and HMBC and comparison with literature. Chemical shifts (δ) in ppm. s, singlet; d, doublet; t, triplet, m, multiplet, dd, doublet of doublet; q, quartet.

**FIGURE 5:**
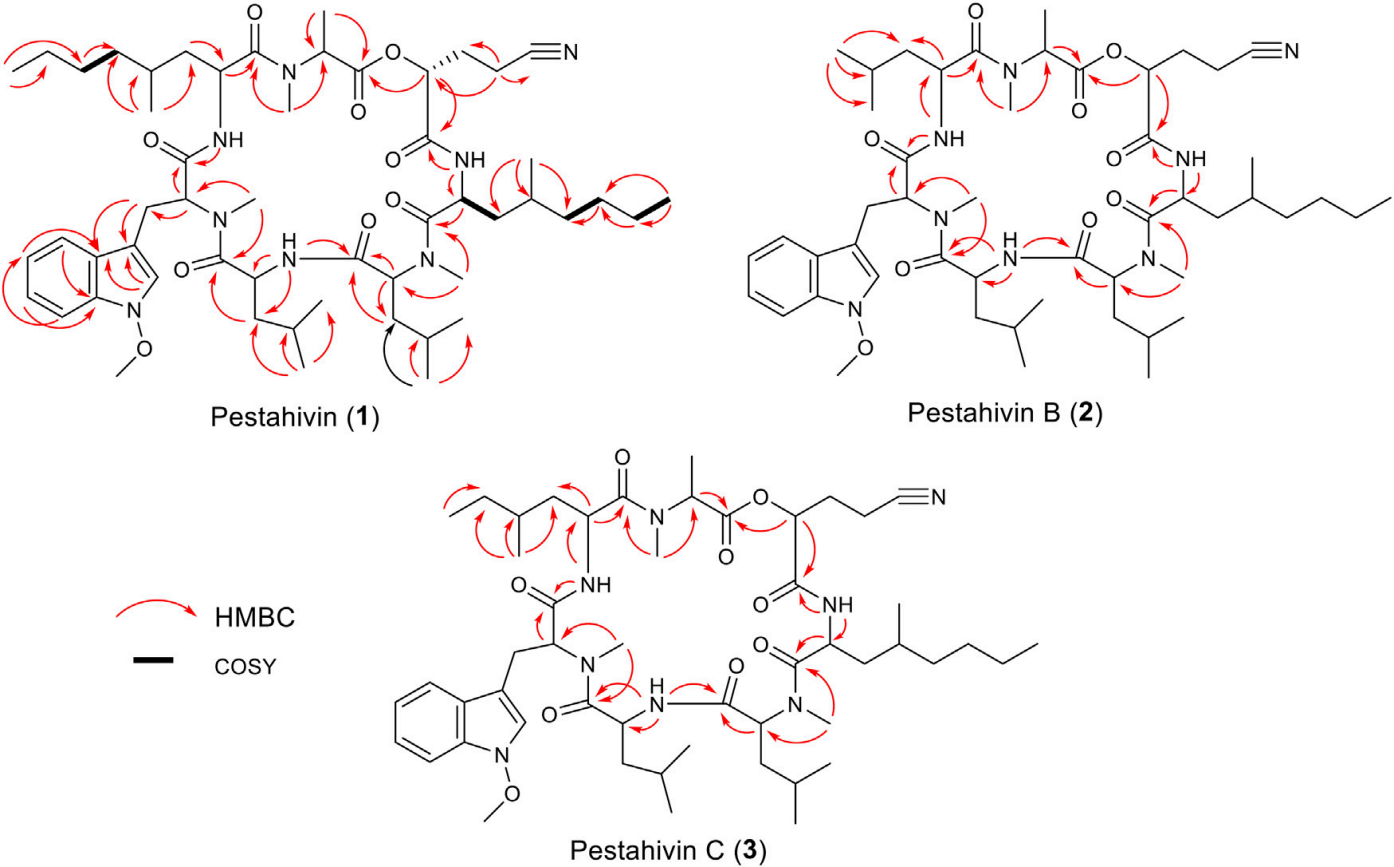
Selected COSY and HMBC correlations for pestahivin (**1**), pestahivins B (**2**) and C (**3**).

**FIGURE 6:**
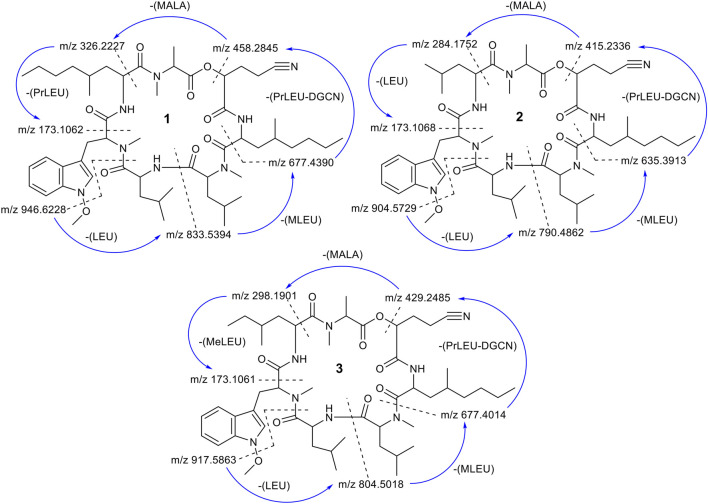
Scheme of the fragmentation pathways of compounds **1**–**3**. The *m/z* ratios were calculated and then compared with the MS^2^ fragment ions. The fragmentation started from the loss of a *O*-methoxy unit followed by a cleavage of the amide bond between residues LEU and MT-O.

Compound **2** was isolated as white amorphous powders and assigned the molecular formula C_50_H_78_N_8_O_9_ following (+)-HRESIMS data analysis (Figure 4). Comparison of NMR and MS data between **1** and **2** suggested that the latter compound was missing a PrLEU residue. Instead, it was replaced with a LEU residue, indicated as LEU-7 in compound **2** following analysis of COSY, HSQC and HMBC spectra of **2** (Figure 5 and Supplementary Figures S12–S16). The connectivity between LEU-7 and MTO-6 was supported by the HMBC correlation from the amide proton (δ_H_ 8.62) of LEU-7 to carbonyl carbon (δ_C_ 167.7) of MTO-6 while the connection between MALA-1 and LEU-7 was deduced from the HMBC correlation between the N-methyl protons (δ_H_ 3.18) of MALA-1 and carbonyl carbon (δ_C_ 170.3) of LEU-7 (Table 3 and Figure 6). Literature review revealed that compound **2** has been previously synthesized by Chen et al. (2002). The ^1^H NMR data of compound **2** (Supplementary Figure S22) were consistent with the literature values of the synthetic version. Thus, the structure of **2** was established and given the name pestahivin B (**2**). Notably, this is the first report of isolation of **2** from a natural source.

**TABLE 3:** NMR spectral data of pestahivins B (**2**) and C (**3**).

Amino acid residue	Position	2^*a*^		3^*b*^	
		^13^C	^1^H, mult. (*J* = Hz)	^13^C	^1^H, mult. (*J* = Hz)
N-Me-Ala (MALA-1)	N-Me	36.6, CH_3_	3.18, s	36.7, CH_3_	3.19, s
	α	58.3, CH	3.95, q (6.8)	58.3, CH	3.97, q (6.8)
	β	13.3, CH_3_	1.37, d (6.7)	13.4, CH_3_	1.37, d (6.8)
	CO	169.9, C		170.0, C	
(*R*)-2-hydroxy-4-cyanobutyric acid (DGCN-2)	1	73.5, CH	4.90, m	73.6, CH	4.88, dd (2.8, 10.9)
	2	26.3, CH_2_	1.71, m; 2.00, m	26.4, CH_2_	1.70, m; 1.99, m
	3	13.1, CH_2_	2.42, m; 2.46, m	13.2, CH_2_	2.45, m; 2.45, m
	4	120.6, CN		120.7, CN	
	CO	167.2, C		167.3, C	
(4*R*)-5-propyl-L-leucine (PrLEU-3)	N-H		7.84, d (9.6)		7.84, d (9.6)
	1	46.4, CH	4.68, q (7.0)	46.5, CH	4.67, q (6.9)
	2	39.3, CH_2_	1.49, m; 1.49, m	39.4, CH_2_	1.50, m; 1.50, m
	3	28.1, CH	1.28, m	28.1, CH	1.28, m
	3-Me	19.9, C	0.80, d (6.4)	20.0, C	0.80, d (6.3)
	4	36.3, CH_2_	1.09, m; 1.19, m	36.3, CH_2_	1.09, m; 1.18, m
	5	28.5, CH_2_	1.17, m; 1.27, m	28.6, CH_2_	1.17, m; 1.27, m
	6	22.3, CH_2_	1.25, m; 1.25, m	22.4, CH_2_	1.24, m; 1.24, m
	7	13.9, CH_3_	0.85, t (6.8)	14.0, CH_3_	0.86, t (6.9)
	CO	169.8, C		169.9, C	
N-Me-Leu (MLEU-4)	N-Me	27.8, CH_3_	2.40, s	27.9, CH_3_	2.39, s
	α	57.3, CH	4.10, m	57.3, CH	4.08, m
	β	35.9, CH_2_	1.57, m; 1.64, m	35.9, CH_2_	1.58, m; 1.64, m
	γ	24.3, CH	1.36, m	24.4, CH	1.37, m
	δ	21.5, CH_3_	0.94, d (6.7)	21.6, CH_3_	0.94, d (7.1)
	δ’	23.3, CH_3_	0.92, d (6.9)	23.3, CH_3_	0.92, d (7.5)
	CO	171.5, C		171.5, C	
Leu (LEU-5)	N-H		8.35, d (6.3)		8.33, d (6.2)
	α	47.3, CH	4.10, m	47.4, CH	4.09, m
	β	36.5, CH_2_	−0.70, m; 1.40, m	36.5, CH_2_	−0.71, m; 1.39, m
	γ	22.7, CH	1.23, m	22.8, CH	1.22, m
	δ	18.0, CH_3_	−0.19, d (6.6)	18.1, CH_3_	−0.19, d (6.6)
	δ'	22.5, CH_3_	0.40, d (6.4)	22.5, CH_3_	0.39, d (6.4)
	CO	172.6, C		172.7, C	
*N*’-methoxy-*N*-methyl-L-tryptophan (MTO-6)	N-Me	28.4, CH_3_	2.79, s	28.6, CH_3_	2.79, s
	1	60.3, CH	4.84, m	60.3, CH	4.83, dd (3.4, 10.6)
	2	23.0, CH_2_	3.04, m; 3.13, m	23.1, CH_2_	3.03, m; 3.11, m
	3	106.1, C		106.2, C	
	4	122.8, CH	7.34, s	122.9, CH	7.36, s
	5-OMe	65.7, CH_3_	4.01, s	65.8, CH_3_	4.01, s
	6	131.7, C		131.7, C	
	7	123.3, C		123.4, C	
	8	118.5, CH	7.62, d (8.0)	118.6, CH	7.62, d (8.0)
	9	119.6, CH	7.07, t (7.6)	119.7, CH	7.07, t (7.8)
	10	122.3, CH	7.19, t (7.5)	122.4, CH	7.20, t (7.3)
	11	108.3, CH	7.42, d (8.2)	108.4, CH	7.43, d (8.1)
	CO	167.7, C		167.9, C	
Leu (LEU-7 in **2**)	N-H		8.62, d (10.0)		
	α	46.2, CH	4.88, m		
	β	39.3, CH_2_	1.31, m; 1.44, m		
	γ	23.6, CH	1.31, m		
	δ	21.8, CH_3_	0.89, d (6.0)		
	δ'	23.0, CH_3_	0.87, d (6.5)		
	CO	170.3, C			
(4*R*)-5-methyl-L-leucine (MeLEU-7 in **3**)	N-H				8.62, d (10.0)
	1			46.2, CH	4.90, q (5.1)
	2			37.9, CH_2_	1.29, m; 1.50, m
	3			29.9, CH	1.11, m
	3-Me			18.3, CH_3_	0.87, d (5.9)
	4			29.4, CH_2_	1.10, m; 1.39, m
	5			11.2, CH_3_	0.82, t (6.8)
	CO			170.5, C	

^*a*1^H (400 MHz) and ^13^C (100 MHz) in DMSO-*d*_6_:chloroform-*d* (10:1).

^*b*1^H (400 MHz) and ^13^C (100 MHz) in DMSO-*d*_6_. Assignments based on COSY, HSQC and HMBC. Chemical shifts (δ) in ppm. s, singlet; d, doublet; t, triplet, m, multiplet, dd, doublet of doublet; q, quartet.

Compound **3** was isolated as yellow amorphous powders. (+)-HRESIMS measurement established the molecular formula C_51_H_80_N_8_O_9_. Comparison of NMR and MS data between **1** and **3** suggested that the latter natural product was missing a PrLEU residue, and this residue was replaced with an unnatural amino acid residue, MeLEU (Figure 6). The MeLEU residue was determined to be MeLEU-7 in compound **3** as supported by the HMBC correlation from the amide proton at δ_H_ 8.62 of MeLEU-7 to carbonyl carbon (δ_C_ 167.9) of MTO-6 in addition to the HMBC correlation between the N-methyl protons (δ_H_ 3.19) of MALA-1 and carbonyl carbon (δ_C_ 170.5) of MeLEU-7 (Table 3 and Figure 5). The structure of **3** was established as a new congener of pestahivin, i.e., pestahivin C. The structure of **3** was very similar with those of **1** and **2**, suggesting they were closely related biosynthetically. Based on spectroscopic data comparison and biosynthetic consideration, the relative configuration of the amino acids in **3** was deemed to be identical with those in **1** and **2**. However, the lack of adequate standards and the rotatable nature of the chiral methyl group hindered the configuration determination of MeLEU in **3**.

To further confirm the structures of compounds **1**–**3**, MS^2^ fragmentation data of compounds **1**–**3** were analyzed and the fragmentation pattern for each compound were proposed. The fragmentation pathway of compounds **1**–**3** started with loss of a *O*-methoxy unit of 31.0184 Da followed by a ring opening (cleavage of the amide bond between residues LEU and MTO) (Figure 6 and Supplementary Figures S4–S6). After the ring opened, the fragment ion *m/z* 946.6228 (calc. *m/z* 946.6256) of **1** continued to lose a unit of LEU (113.0841 Da). The sequential loss of a unit of Leu explained the occurrence of fragment ion *m/z* 833. 5,394 (calc. *m/z* 833.5415). This is followed by the sequential loss of a unit of MLEU with 155.0946 Da that explained the occurrence of fragment ion *m/z* 677.4390 (calc. *m/z* 677.4390). The continued loss of a unit of PrLEU-DGCN with 222.1732 Da led to the formation of a fragment ion *m/z* 458.2845 (calc. *m/z* 458.2893). The subsequent loss of a unit of MALA with 130.0504 Da and a unit of PrLEU with 155.1310 Da explained the occurrence of the final fragment ion of MTPO *m/z* 173.1062 (calc. *m/z* 173.1079). This fragment ion resulted from a neutral loss of a C=O. In total, the fragmentation pathway of compounds **1**–**3** took four major steps of losing a unit of LEU, a unit of MLEU, a unit of PrLEU-DGCN and a unit of MALA. However, since compounds **2** and **3** has different substitution at the seventh residue, the last fragmentation step is the losing of a unit of LEU with 113.0841 Da in compound **2** and a unit of MeLEU with 127.0997 Da in compound **3** (Supplementary Figures S5, S6). In addition, MS/MS molecular network analysis (Supplementary Figure S27) further confirmed the structural correlations between compounds **1**–**3**.

### 3.5 Chemical structural data

The NMR spectra of pestahivins (**1**) and pestahivin B (**2**) and C (**3**) are provided in Supplementary Figures S7–S25.

#### 3.5.1 Pestahivin (1)

White amorphous powders; ${\left[\alpha \right]}_{D}^{23}$ − 41 (c 0.07, MeOH); UV (MeOH) λ_max_ 224 nm; HR-ESI-MS *m/z* 977.6439 [M+H]^+^ (calcd for C_53_H_84_N_8_O_9_ + H, 977.6434), see Supplementary Figure S26; ^1^H and ^13^C NMR data, see Table 2.

#### 3.5.2 Pestahivin B (2)

White amorphous powders; ${\left[\alpha \right]}_{D}^{23}$ −29 (c 0.7, MeOH); UV (MeOH) λ_max_ 224 nm; HR-ESI-MS *m/z* 935.5976 [M+H]^+^ (calcd for C_50_H_78_N_8_O_9_ + H, 935.5965), see Supplementary Figure S26; ^1^H and ^13^C NMR data, see Table 3.

#### 3.5.3 Pestahivin C (3)

Yellow amorphous powders; ${\left[\alpha \right]}_{D}^{23}$ −0.26 (c 0.4, MeOH); UV (MeOH) λ_max_ 224 nm; HR-ESI-MS *m/z* 949.6120 [M+H]^+^ (calcd for C_51_H_80_N_8_O_9_ + H, 949.6121), see Supplementary Figure S26; ^1^H and ^13^C NMR data, see Table 3.

### 3.6 Antimicrobial and cytotoxic activities of pestahivin and its analogues

Pestahivin (**1**) and its new analogues pestahivins B (**2**) and C (**3**) isolated from *Bartalinia* sp. F9447 were subjected to antimicrobial activity evaluation against *S. aureus*, *C. albicans* and *A. brasiliensis* and cytotoxicity testing against A549, MIA PaCa-2 and PANC-1 cell lines. Pestahivin (**1**) and its analogue C (**3**) showed similar antifungal activity profiles against *C. albicans* with IC_50_ value of 1.9 ± 0.26 µM, and similar activity against *A. brasiliensis* with IC_50_ values of 0.46 ± 0.06 and 0.65 ± 0.07 µM, respectively (Table 4, Supplementary Figure S28). The observed differences in the antimicrobial and cytotoxic activities between compounds was statistically significant as demonstrated by one-way analysis of variance (ANOVA). Pestahivin analogue C (**3**) showed 45-fold and 7-fold more potent IC_50_ against *C. albicans* and *A. brasiliensis* respectively, when compared to pestahivin B (**2**). No antibacterial activity against *S. aureus* was observed for all three compounds. All three compounds demonstrated more potent cytotoxic activity against MIA PaCa-2 than A549 cells. There was no significant inhibition against PANC-1 pancreatic cell line, where the inhibitory activity plateaued at 20–30% inhibition. The dose-response curves for the three compounds are shown in Supplementary Figure S28. Pestahivin was isolated for the first time from *Pestalotiopsis* sp. RF-5890 strain and was found to exhibit a strong antiviral effect against the human immunodeficiency virus (HIV) (Itazaki et al., 1995). Interestingly, there was no other literature reporting the isolation of pestahivin or related compound from Nature, which makes the discovery of pestahivin and its two novel analogues in this study an interesting finding. It is worthy to note, that *Bartalinia* sp. F9447 and *Pestalotiopsis* sp. RF-5890 belong to the same family of *Sporocadaceae*.

**TABLE 4:** Comparison of the bioactivity of the three isolated compounds with the reference standard compounds used in the study.

Test compound	IC_50_ and IC_90_ values for compounds 1–3 (µM)								
	Antifungal assay			Antibacterial assay			Cytotoxicity assay		
	CA IC_50_	CA IC_90_	AB IC_50_	AB IC_90_	SA IC_50_	SA IC_90_	A549 IC_50_	MIA PaCa-2 IC_50_	PANC-1 IC_50_
Pestahivin (**1**)	1.9 ± 0.26	5.6 ± 1.1	0.46 ± 0.06	1.4 ± 0.38	> 100	> 100	1.0 ± 0.27	0.65 ± 0.12	> 100
Pestahivin B (**2**)	89 ± 17	144 ± 18	4.5 ± 0.48	33 ± 5.8	> 100	> 100	42 ± 5.2	6.2 ± 6.1	> 100
Pestahivin C (**3**)	1.9 ± 0.16	3.4 ± 1.5	0.65 ± 0.07	1.5 ± 0.16	> 100	> 100	17.8 ± 9.7	5.5 ± 0.90	> 100
Gentamicin	−	−	−	−	0.17 ± 0.02	0.34 ± 0.02	−	−	−
Amphotericin B	0.14 ± 0.04	0.16 ± 0.04	0.21 ± 0.02	0.28 ± 0.01	−	−	−	−	−
Puromycin	−	−	−	−	−	−	0.37 ± 0.06	0.38 ± 0.03	0.41 ± 0.04
*p*-value*	0	0	0	0	−	−	0.00001	0.0047	−

*One -way ANOVA test *p* < 0.05.

CA = *C. albicans*, AB = *A. brasiliensis* and SA = *S*. *aureus*.

### 3.7 Taxonomy of bioactive strain F9447

**FIGURE 7:**
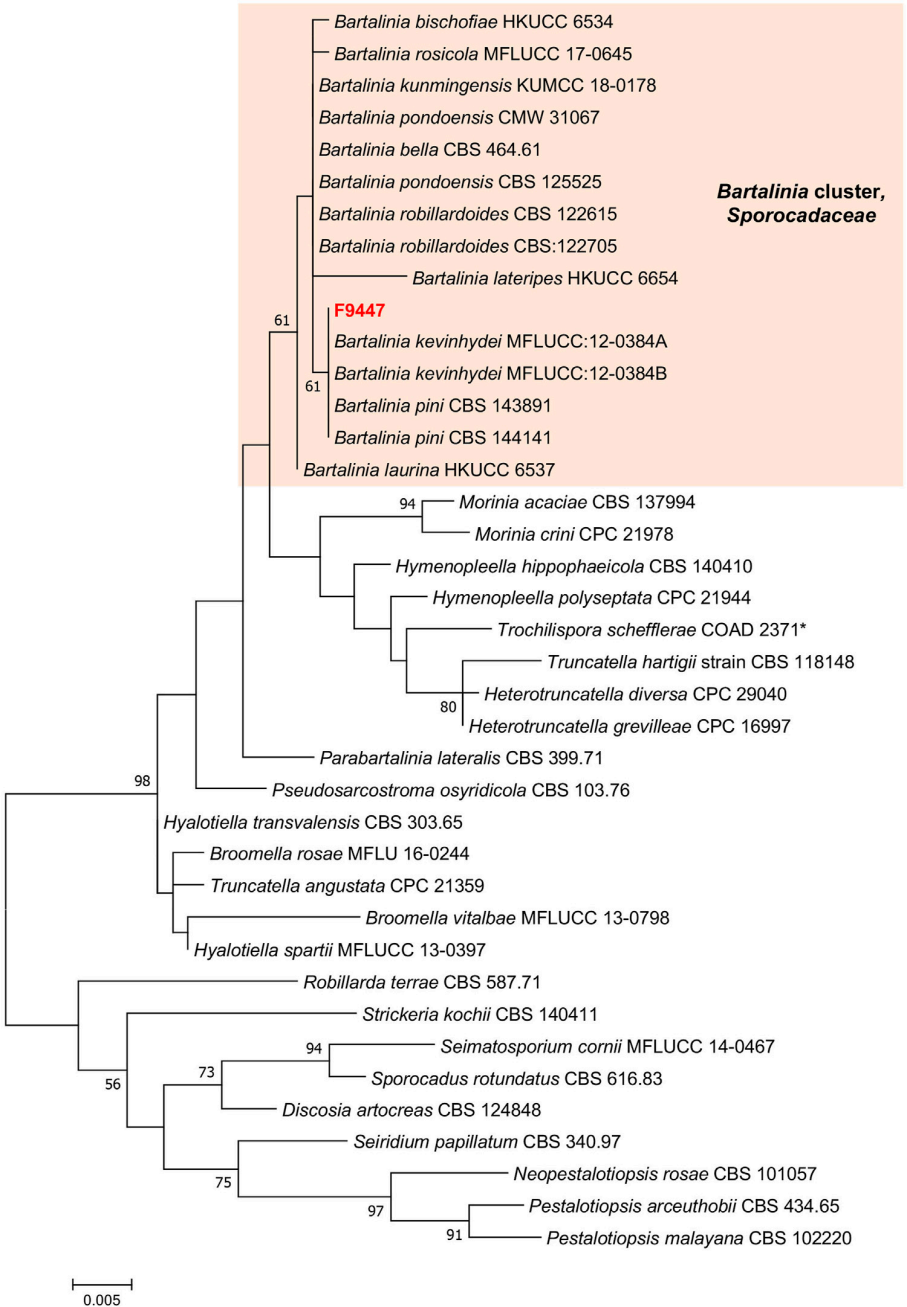
Phylogram generated from maximum likelihood analysis based on LSU, SSU, ITS2, *TEF-1α*, RPB2 and TUB dataset of the strain F9447 and representative species in *Sporocadaceae* family. The evolutionary history was inferred by using the Maximum Likelihood method based on the Tamura-Nei model (Tamura and Nei, 1993). Evolutionary analyses were conducted in MEGA 7. Bootstrap support values for ML equal to or greater than 50% are given above the nodes. *Trochilispora schefflerae COAD 2371 belongs to *Amphisphaeriaceae* family. Strain informations and accession numbers can be found in Supplementary Table S4.

Phylogenetic analyses of a combined LSU, ITS, TEF, RPB2 and *β*-tubulin sequence dataset (Figure 7, Supplementary Table S4) show that strain F9447 cluster with *Bartalinia* species clade and closely related to *B. pini* CBS 143891, *B. kevinhydei* MFLUCC:12–0384A and *B. bella* CBS 464.61. Comparison of LSU and ITS region reveals that strain F9447 is not significantly different from closely related species *B. pini* CBS 143891, *B. kevinhydei* and *B. bella* CBS 464.61 (from 1-4 differentiated nucleotide bases); however, strain F9447 is significantly different from *B. bella* CBS 464.61*, B. pini* CBS 143891 in RPB2 region (18 and 54 nucleotide bases), TEF region (35 and 84 nucleotide bases), and *β*-tubulin region (19 and 52 nucleotide bases). Similarly, a comparison of ITS region shows that previously published reference strain *B. pini* CBS 143891 is not significantly different from *B. robillardoides* CBS 122615 (only 2 differentiated nucleotide bases); however, *B. pini* CBS 143891 is different from *B. robillardoides* CBS 122615 in 43, 81, and 58 nucleotide bases in RPB2, TEF and *β*-tubulin regions respectively. Strain F9447 is therefore a potentially new species based on LSU, ITS, TEF, RPB2 and *β*-tubulin sequence dataset. We therefore conclude that strain F9447 is possibly a new species in *Bartalinia* genus which was isolated from grass flower collected from Sungei Buloh Wetland Reserve, Singapore. This strain was found to produce new pestahivin analogues whose biosynthesis was enhanced in the presence of two chemical elicitors.

## 4 Conclusion

In this study, phylogeny, antimicrobial and cytotoxic potential of endophytic fungi isolated from Sungei Buloh Wetland Reserve, Singapore were investigated. Our findings provide an insight into the species richness of endophytic fungal community from mangrove and other plants growing in this area. Overall, our results reveal the presence of a highly diverse endophytic fungal community in the study area which may include potential novel undescribed strains. All the studied fungal strains were affiliated to the phylum *Ascomycota* with *Colletotrichum* being the most dominant fungal genus. Extracts generated from 23 fungal strains grown in two media with or without two epigenetic modifiers exhibited varied levels of antimicrobial and cytotoxic activities. One fungal strain, *Bartalinia* sp. F9447 exhibited enhanced antifungal activity when grown in the presence of 5-azacytidine. Large-scale fermentation and isolation studies led to the isolation of pestahivin and its two novel analogues. The levels of production of the three pestahivin compounds were greatly enhanced when the fungal strain was grown in the presence of two chemical elicitors. To the best of our knowledge, this is the second time that pestahivin has been isolated from fungi, and the first time that two of its analogues are being reported. The study thus further demonstrates that chemical elicitation using epigenetic modifiers is an important tool for enhanced production of constitutive fungal secondary metabolites or for discovery of new natural products.

## Data Availability

The datasets presented in this study can be found in online repositories. The names of the repository/repositories and accession number(s) can be found in the article/Supplementary Material.
