# Supplementary material for: Chemical elicitation as an avenue for discovery of bioactive compounds from fungal endophytes
Source: Front Chem. 2022 Nov 23;10:1024854. doi: 10.3389/fchem.2022.1024854 (PMC9727085; doi:10.3389/fchem.2022.1024854)
Supplement: Supplementary file 1 [file DataSheet1.docx]

Supplementary Material

**Chemical elicitation as an avenue for discovery of bioactive compounds from fungal endophytes**

Madhaiyan Munusamy^1†^, Kuan Chieh Ching^1†^, Lay Kien Yang^1^, Sharon Crasta^1^, Martin Muthee Gakuubi ^1,2^, Zhao Yan Chee^1^, Mario Wibowo^1^, Chung Yan Leong^1^, Yoganathan Kanagasundaram^1^ and Siew Bee Ng^1^*

^1^Singapore Institute of Food and Biotechnology Innovation (SIFBI), Agency for Science, Technology and Research (A*STAR), Singapore.

^2^School of Biological Sciences, Nanyang Technological University, Singapore.

^†^These authors have contributed equally to this work.

*** Correspondence:**

Siew Bee Ng, E-mail: [ngsb@sifbi.a-star.edu.sg](mailto:ngsb@sifbi.a-star.edu.sg)

KEYWORDS

*Bartalinia* sp.*,* endophytes, chemical elicitors, pestahivin analogues, mangrove.

**
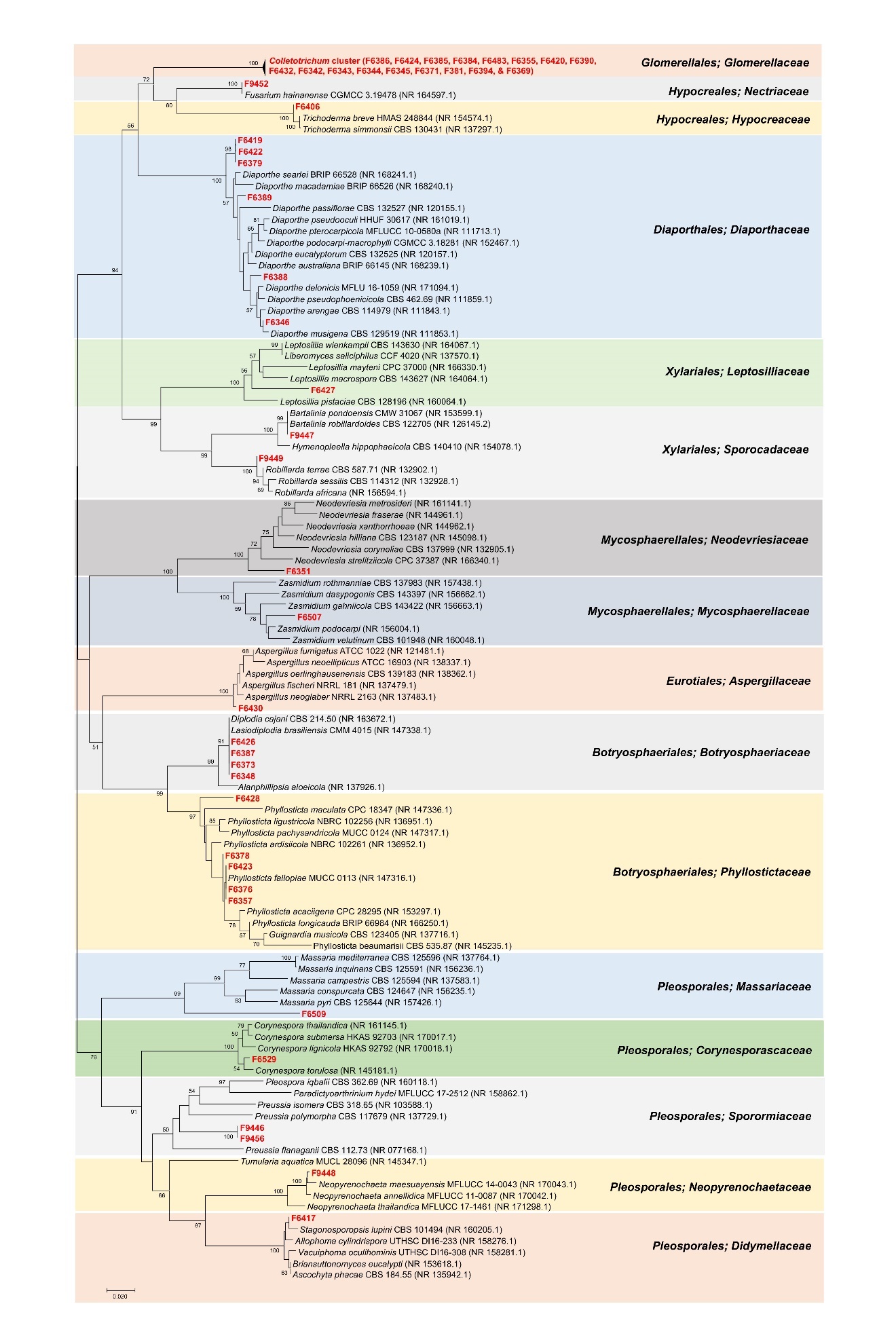
**

FIGURE S1

Phylogenetic analysis of internal transcribed spacer (ITS2) gene sequences of the 46 endophytic fungi from NPL A*STAR. The evolutionary history was inferred using the Neighbor-Joining method (Saitou and Nei, 1987). The optimal tree with the sum of branch length = 2.80627415 is shown. The percentage of replicate trees in which the associated taxa clustered together in the bootstrap test (500 replicates) are shown next to the branches (Felsenstein, 1985). The tree is drawn to scale, with branch lengths in the same units as those of the evolutionary distances used to infer the phylogenetic tree. The evolutionary distances were computed using the Maximum Composite Likelihood method (Tamura et al., 2004) and are in the units of the number of base substitutions per site. The analysis involved 124 nucleotide sequences. Evolutionary analyses were conducted in MEGA7 (Kumar et al., 2016).


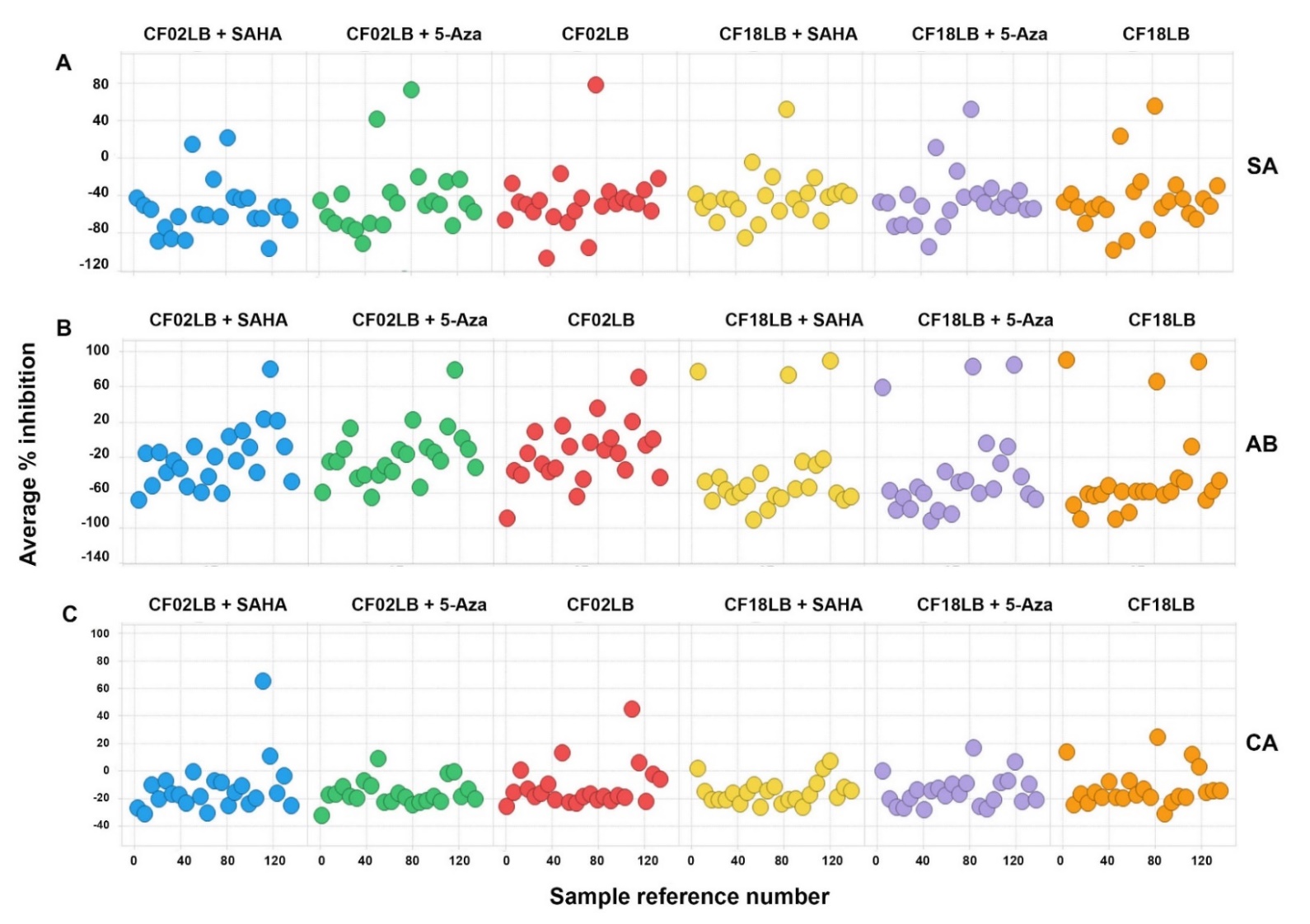


FIGURE S2

Distribution of antibacterial and antifungal hits from 23 fungal strains grown in two media (CF02LB and CF18LB) in the presence and absence of two chemical elicitors; 5-azacytidine (5-Aza) and suberoylanilide hydroxamic acid (SAHA). SA = S. *aureus*, AB = *A. brasiliensis* and CA = *C. albicans*.

**
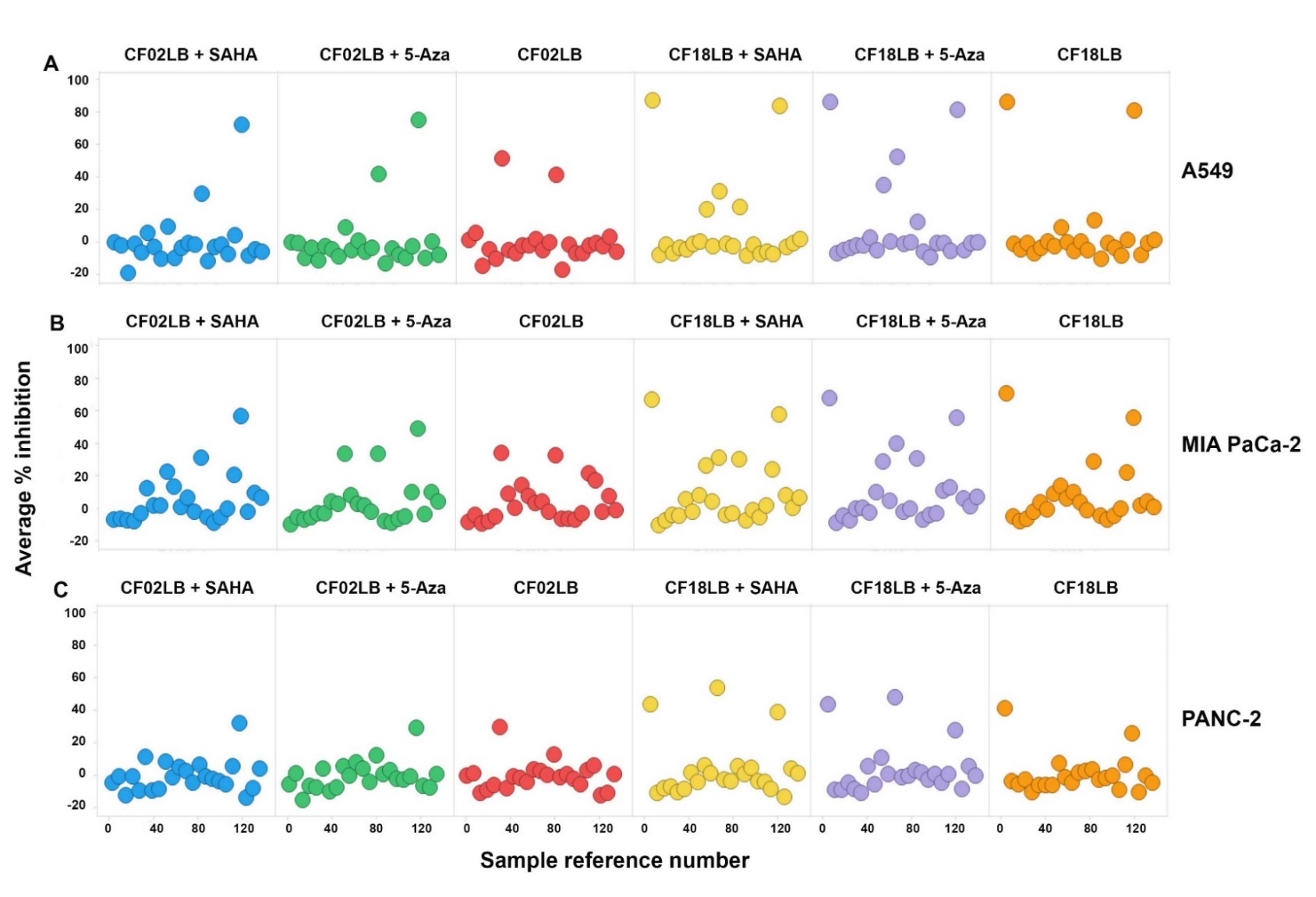
**

FIGURE S3

Distribution of cytotoxic hits from 23 fungal strains grown in two media (CF02LB and CF18LB) in the presence and absence of two chemical elicitors; 5-azacytidine (5-Aza) and suberoylanilide hydroxamic acid (SAHA) against A549, MIA PaCa-2 and PANC-1 cancer cell lines.

**
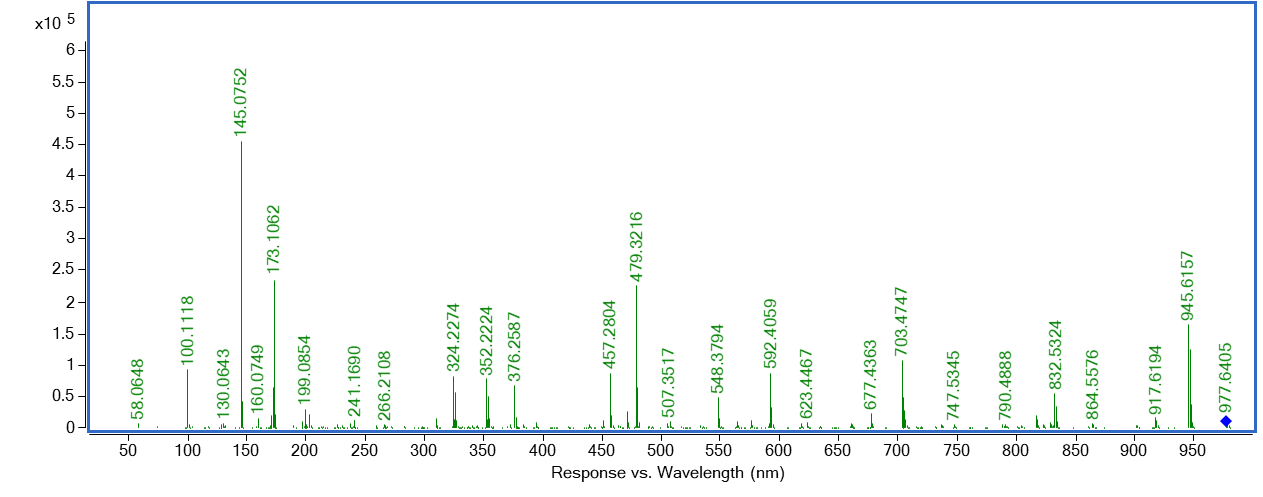
**

FIGURE S4. MS/MS spectrum of pestahivin (**1**) from *m/z* 50 – *m/z* 1000.


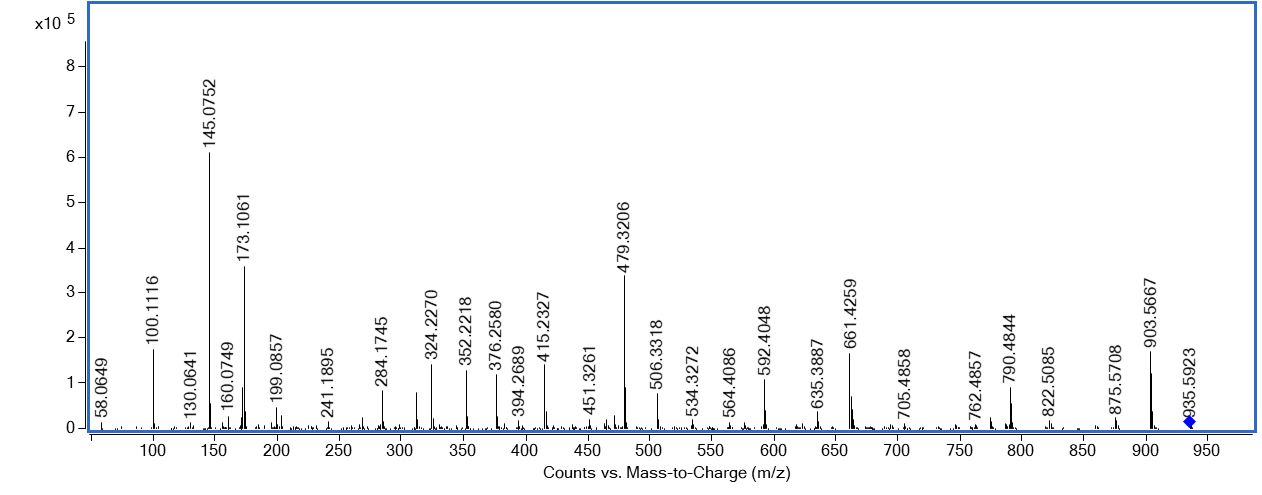


FIGURE S5. MS/MS spectrum of pestahivin B (**2**) from *m/z* 50 – *m/z* 1000.

**
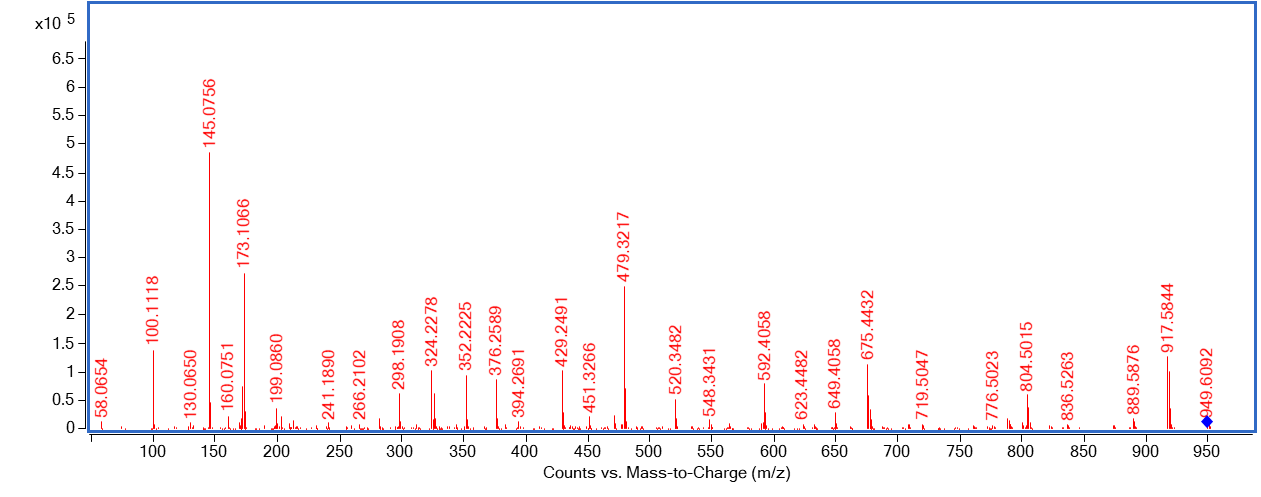
**

FIGURE S6. MS/MS spectrum of pestahivin C (**3**) from *m/z* 50 – *m/z* 1000.


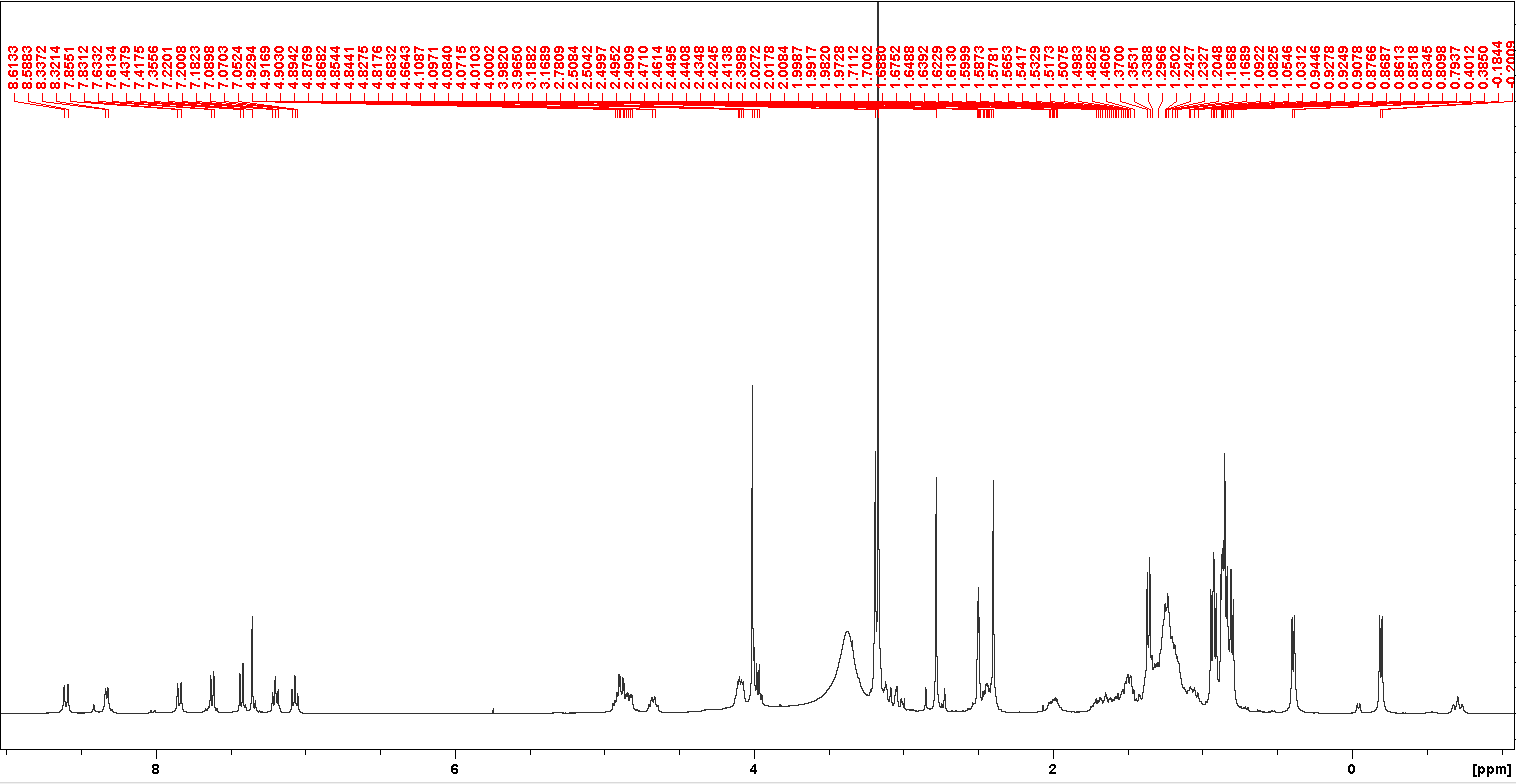


FIGURE S7. ^1^H NMR spectrum (DMSO-*d_6_*, 400 MHz) of pestahivin (**1**).


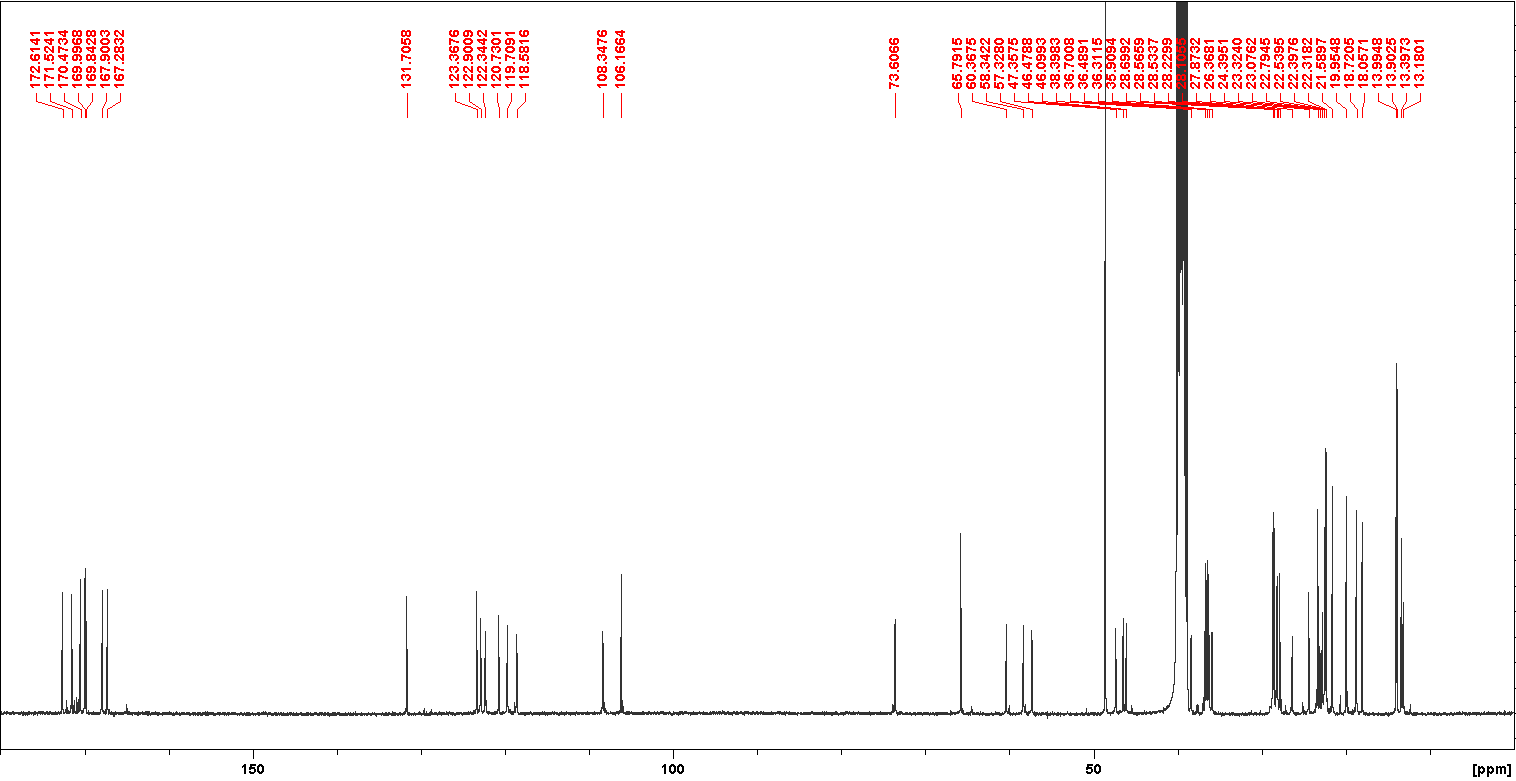


FIGURE S8. ^13^C NMR spectrum (DMSO-*d_6_*, 100 MHz) of pestahivin (**1**).


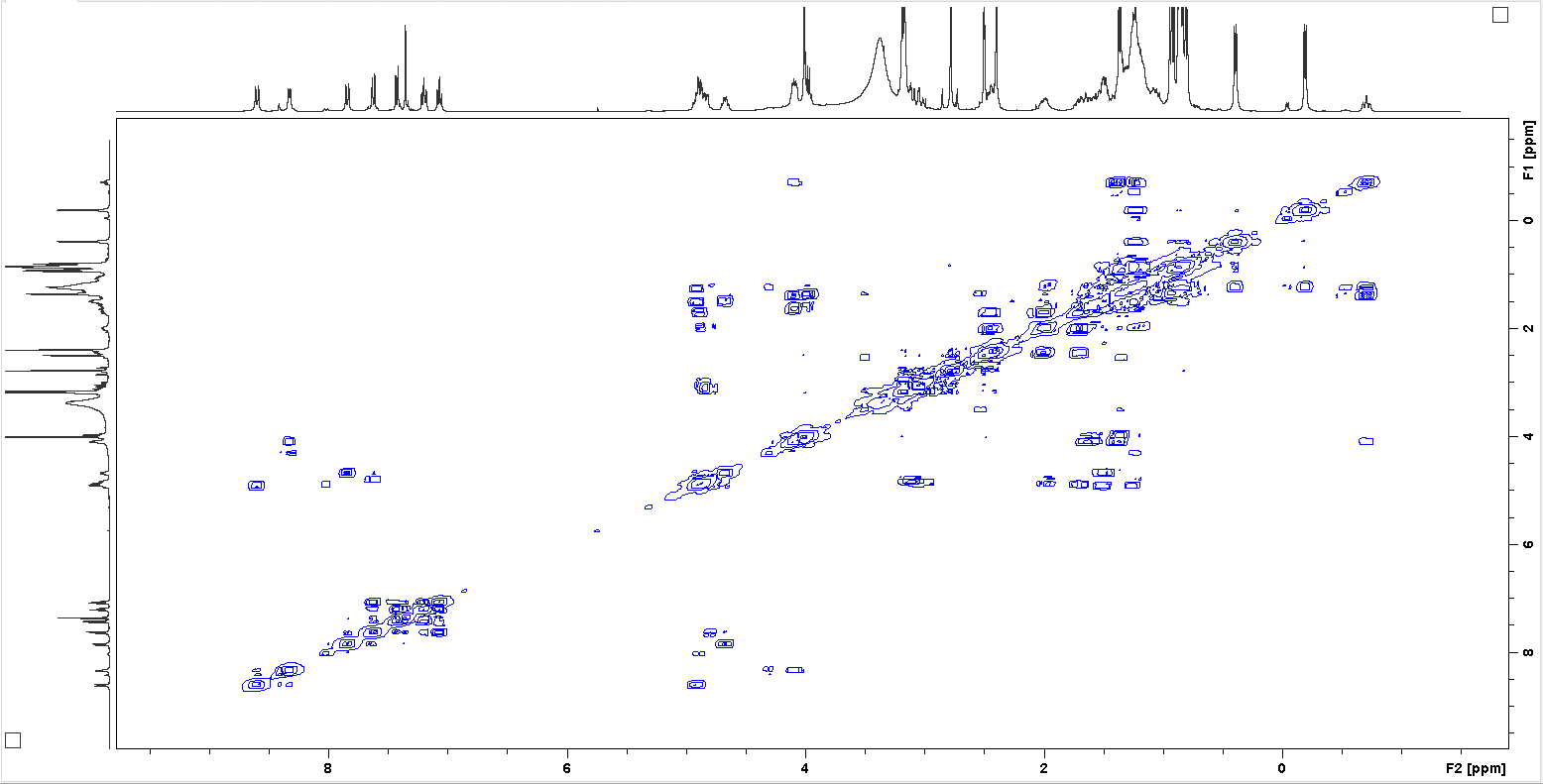


FIGURE S9. COSY spectrum (DMSO-*d_6_*) of pestahivin (**1**).


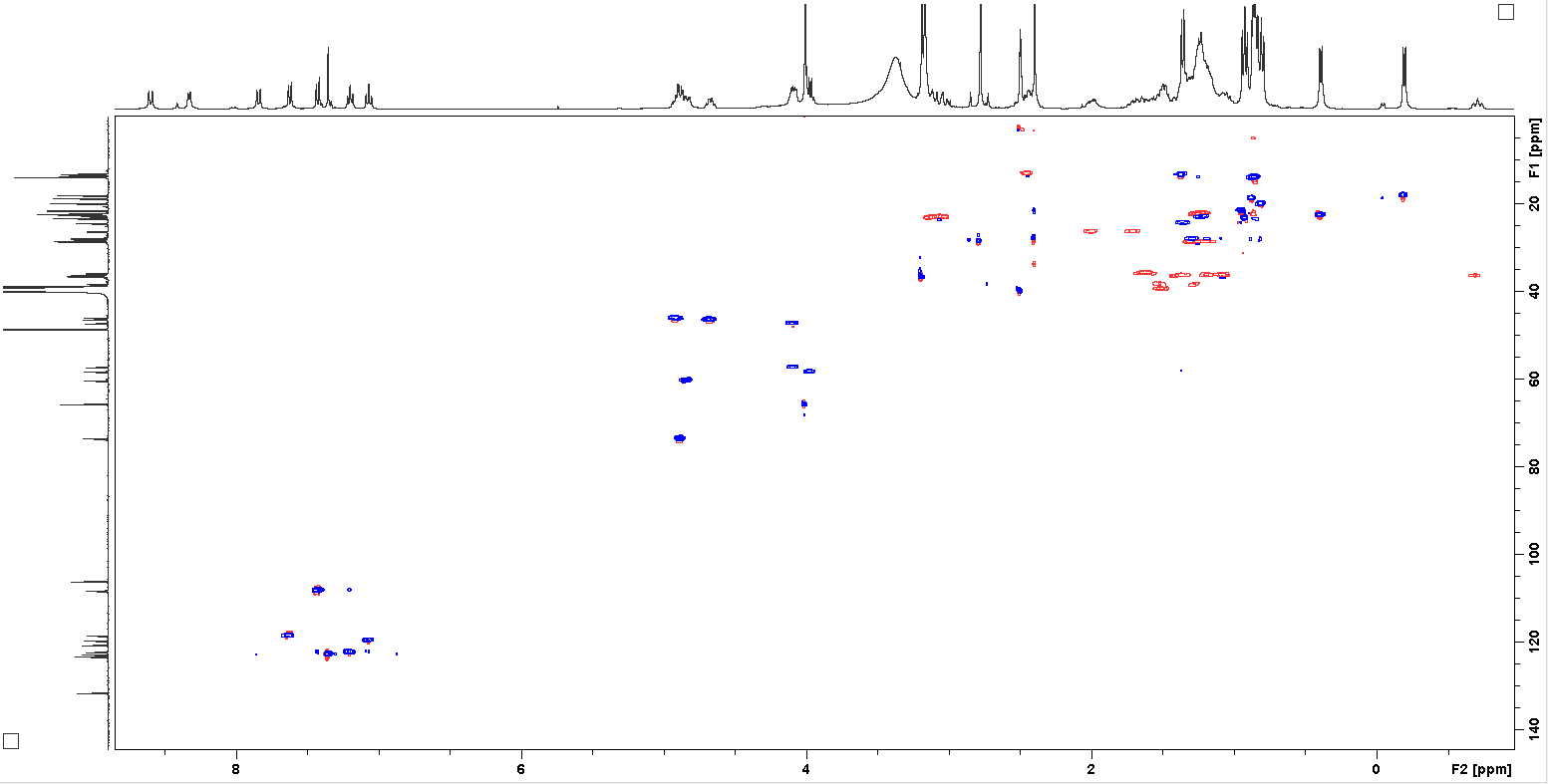


FIGURE S10. HSQC spectrum (DMSO-*d_6_*) of pestahivin (**1**).


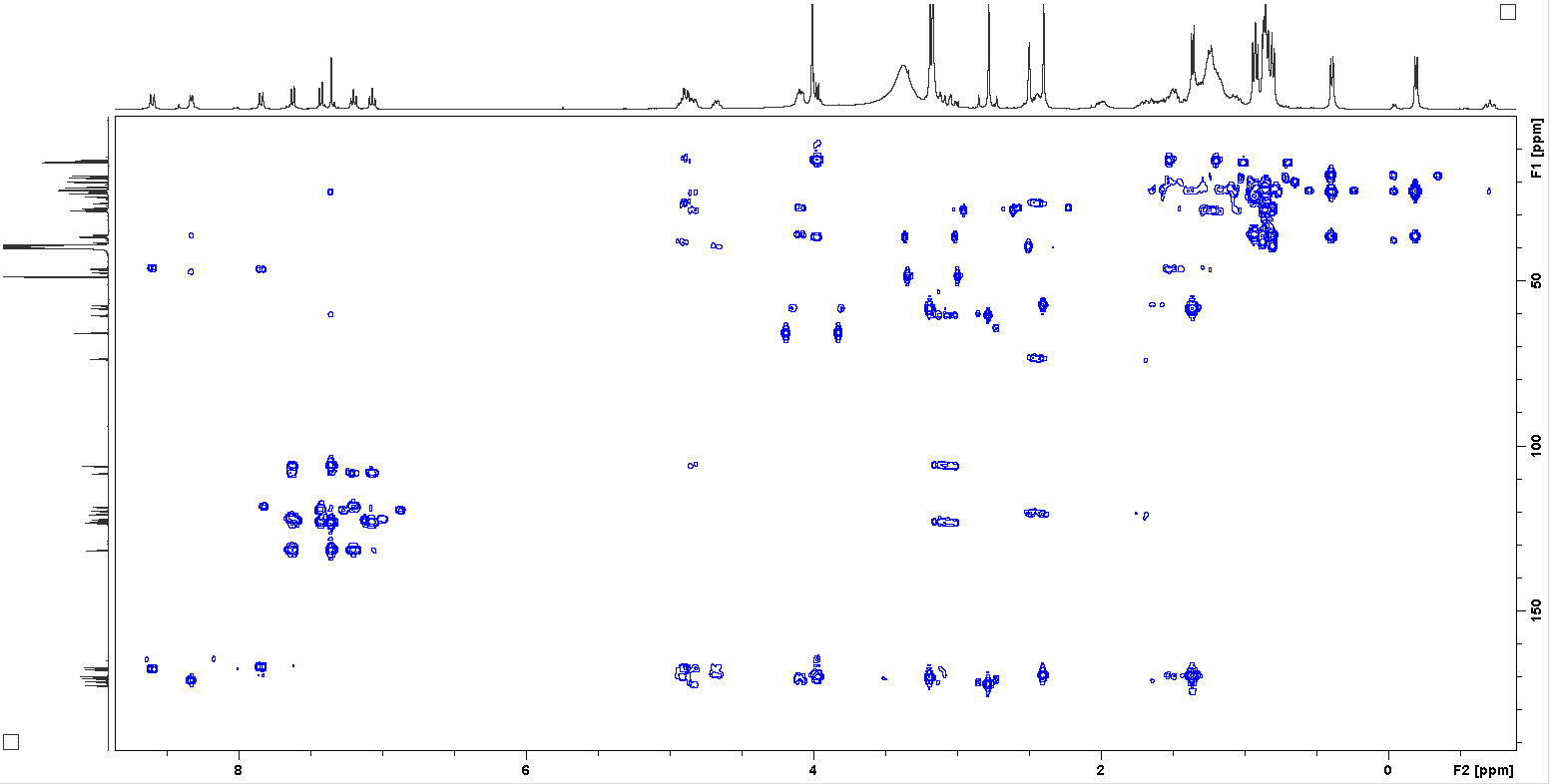


FIGURE S11. HMBC spectrum (DMSO-*d_6_*) of pestahivin (**1**).


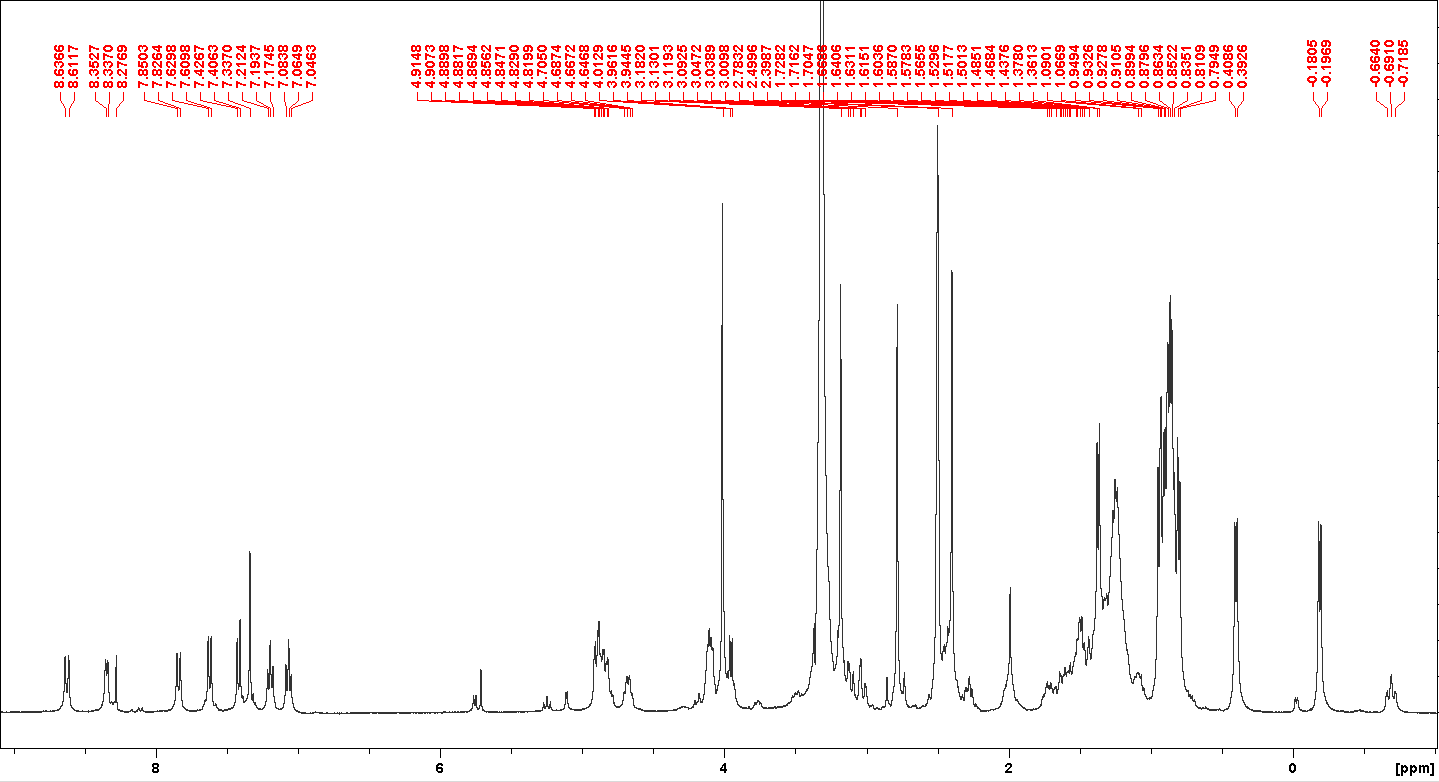


FIGURE S12. ^1^H NMR spectrum (DMSO-*d*_6_:chloroform-*d* (10:1), 400 MHz) of pestahivin B (**2**).


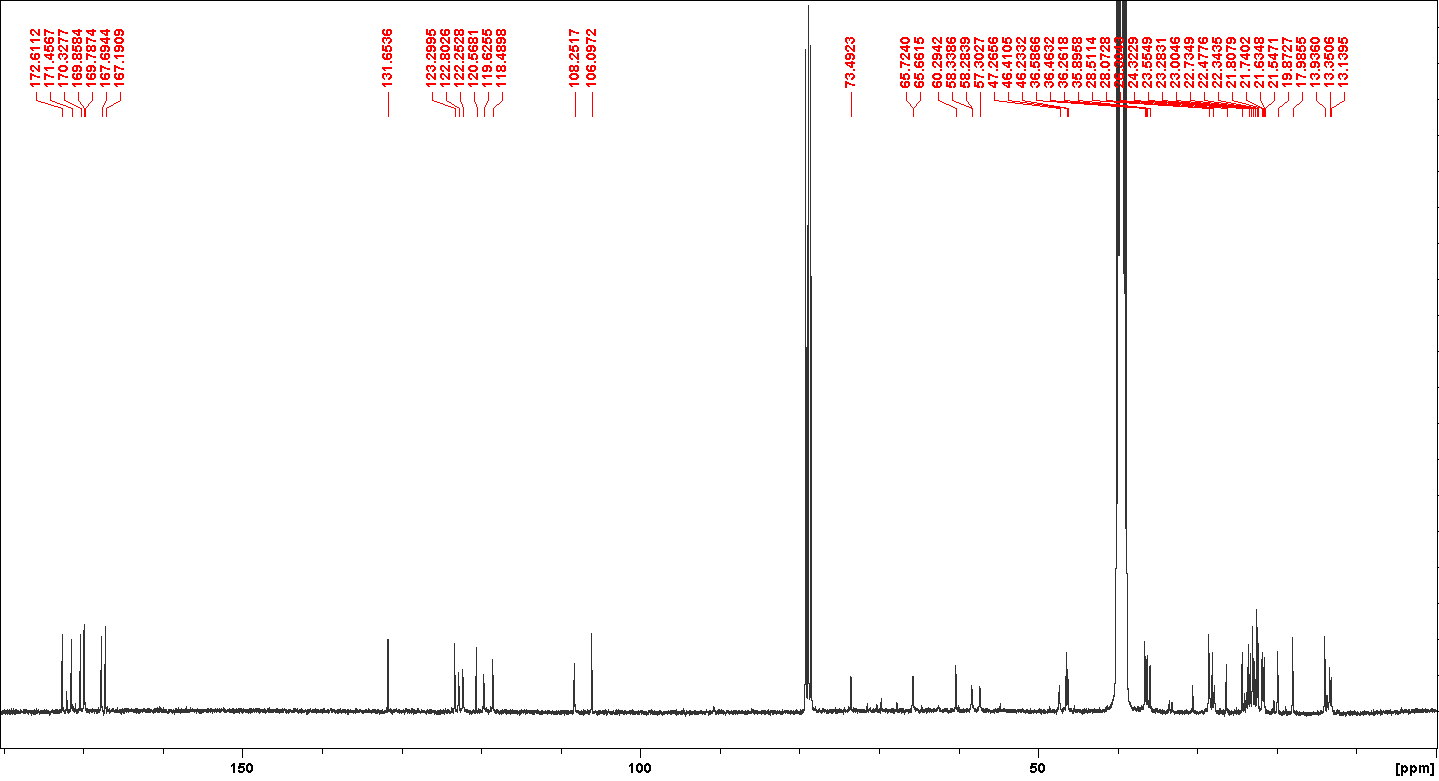


FIGURE S13. ^13^C NMR spectrum (DMSO-*d*_6_:chloroform-*d* (10:1), 100 MHz) of pestahivin B (**2**).


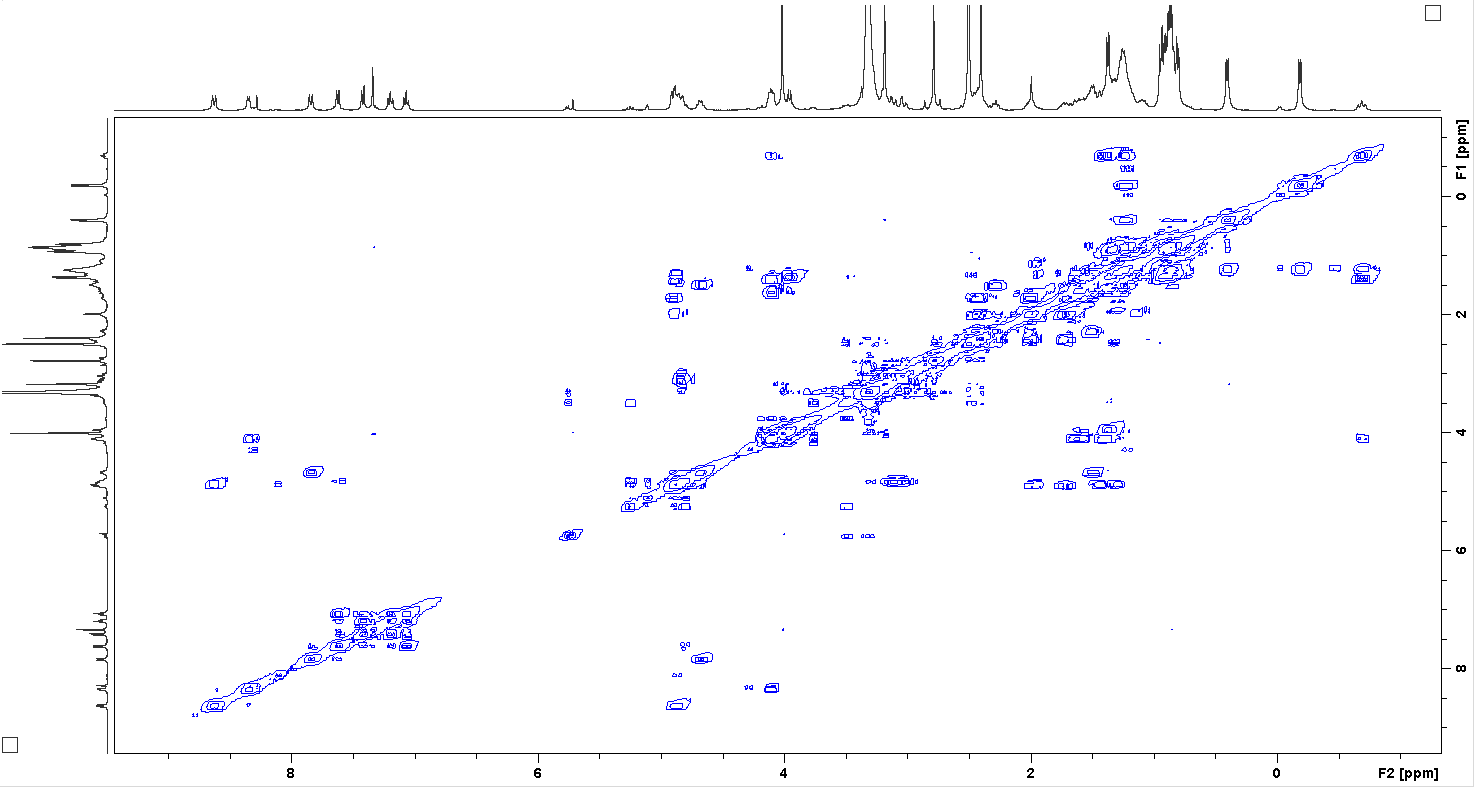


FIGURE S14. COSY spectrum (DMSO-*d*_6_:chloroform-*d* (10:1)) of pestahivin B (**2**).


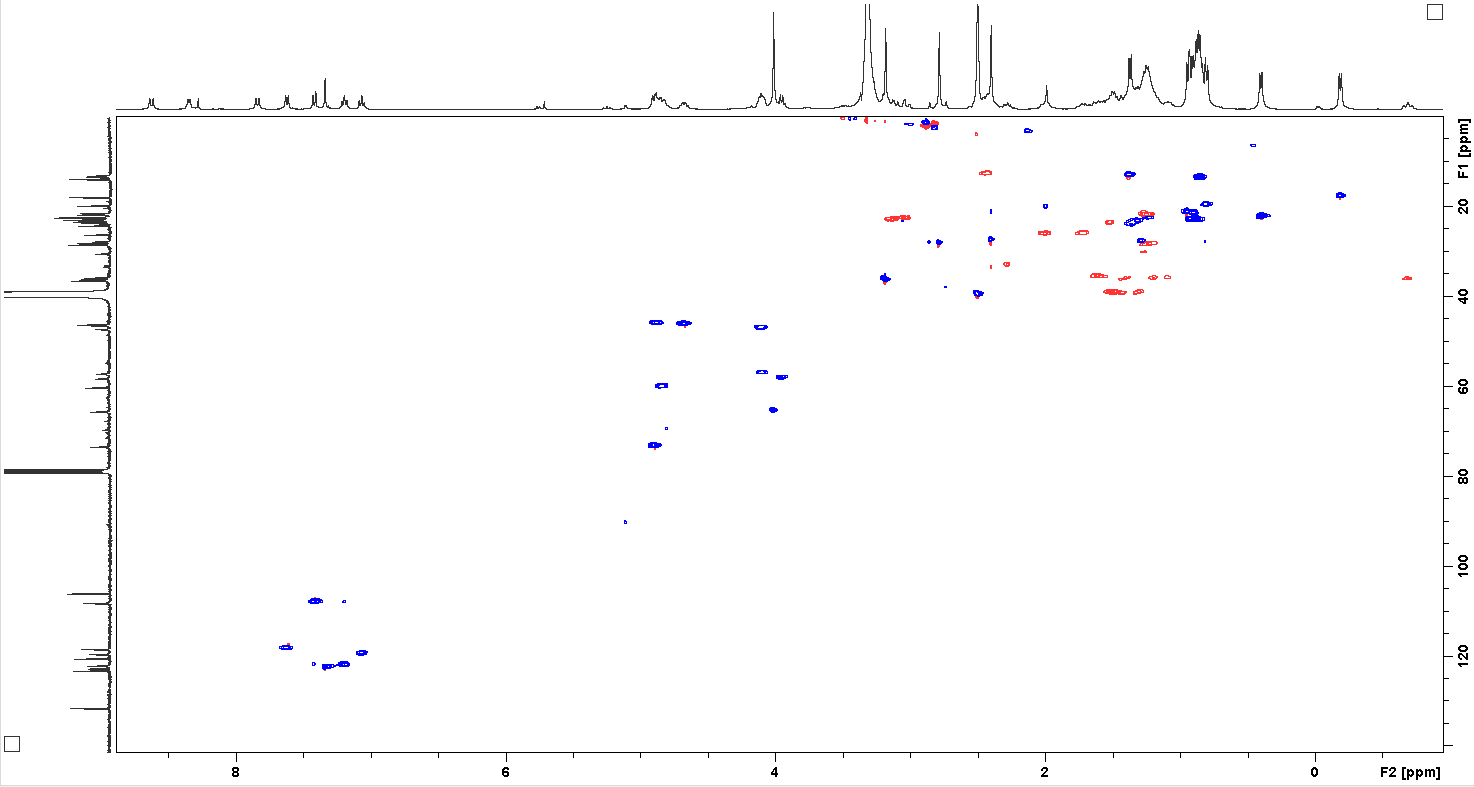


FIGURE S15. HSQC spectrum (DMSO-*d*_6_:chloroform-*d* (10:1)) of pestahivin B (**2**).


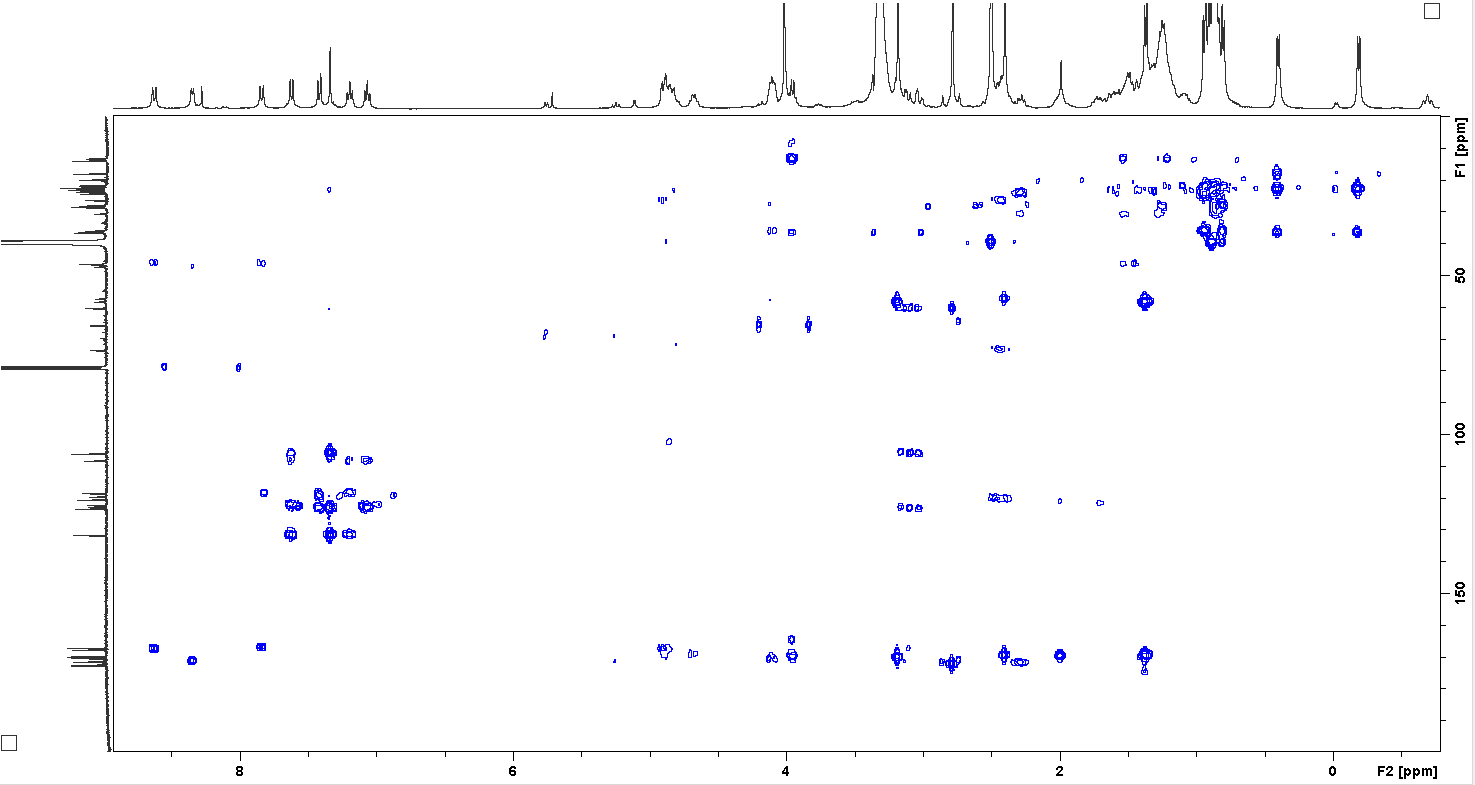


FIGURE S16. HMBC spectrum (DMSO-*d*_6_:chloroform-*d* (10:1)) of pestahivin B (**2**).


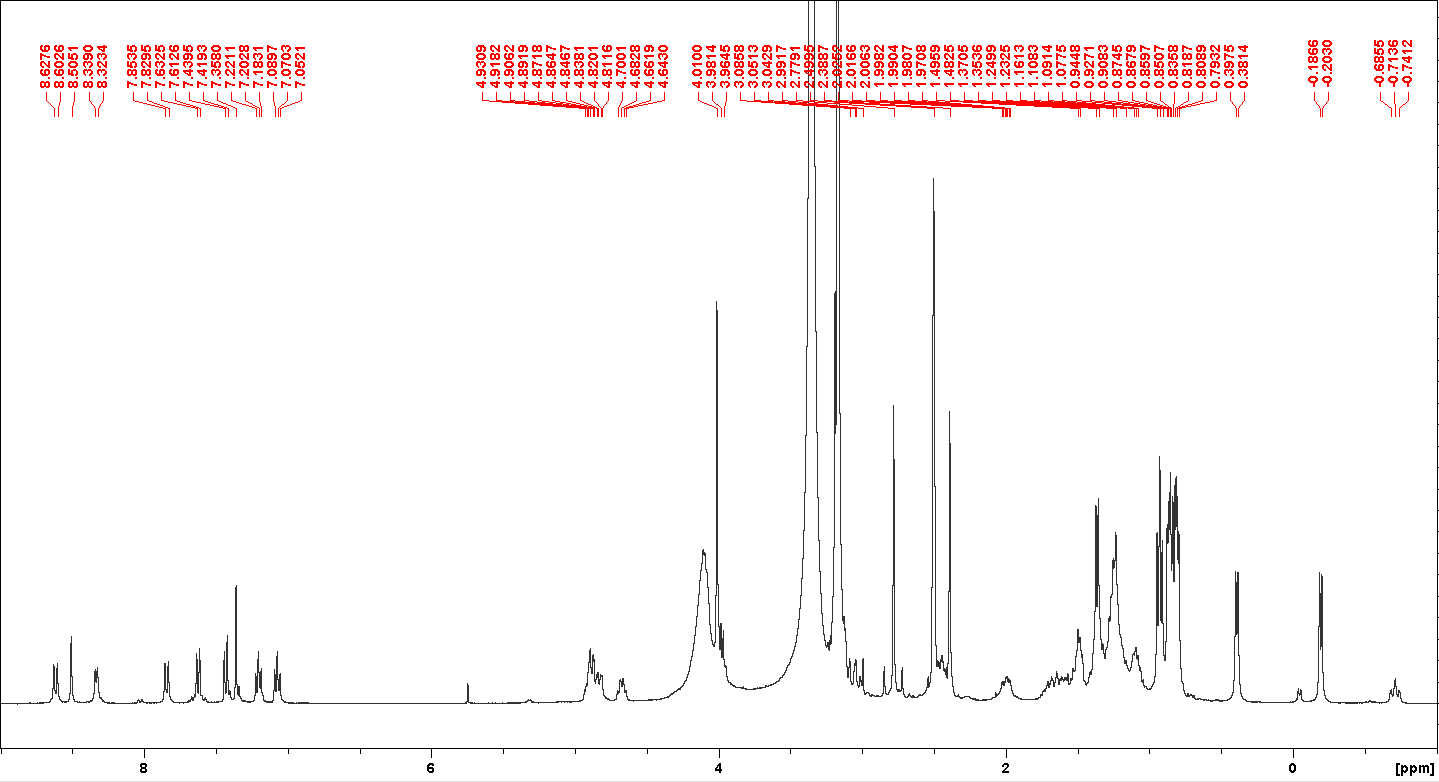


FIGURE S17. ^1^H NMR spectrum (DMSO-*d_6_*, 400 MHz) of pestahivin C (**3**).


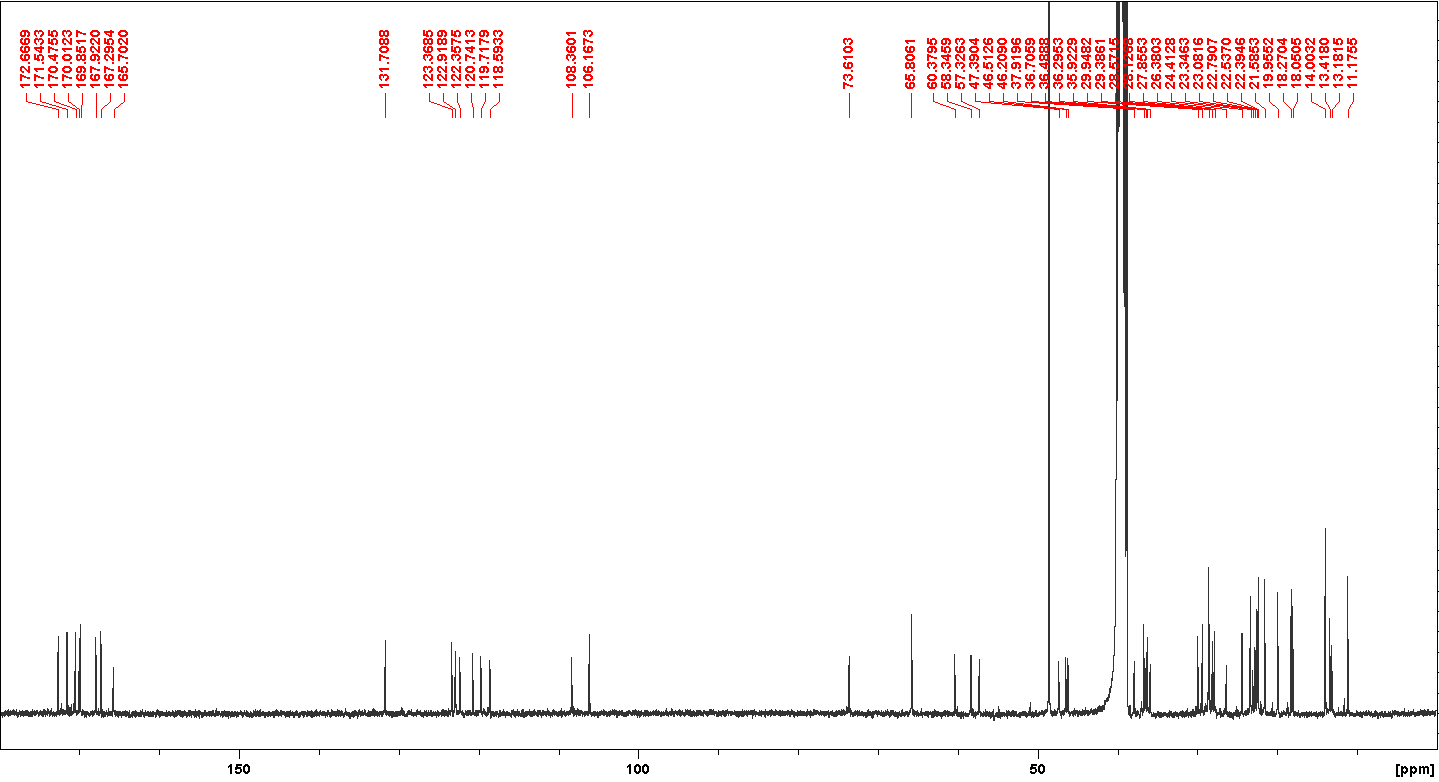


FIGURE S18. ^13^C NMR spectrum (DMSO-*d_6_*, 100 MHz) of pestahivin C (**3**).


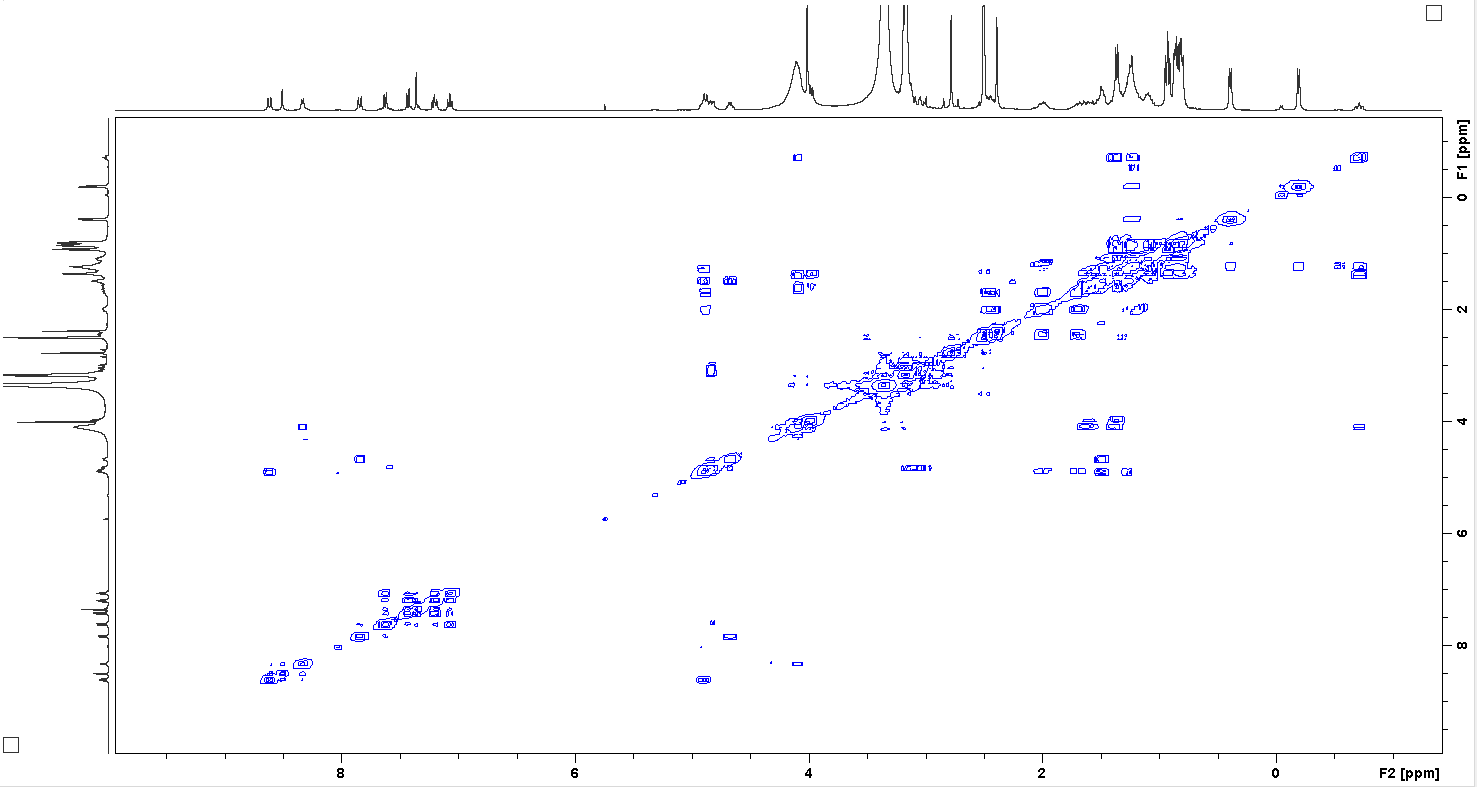


FIGURE S19. COSY spectrum (DMSO-*d_6_*) of pestahivin C (**3**).


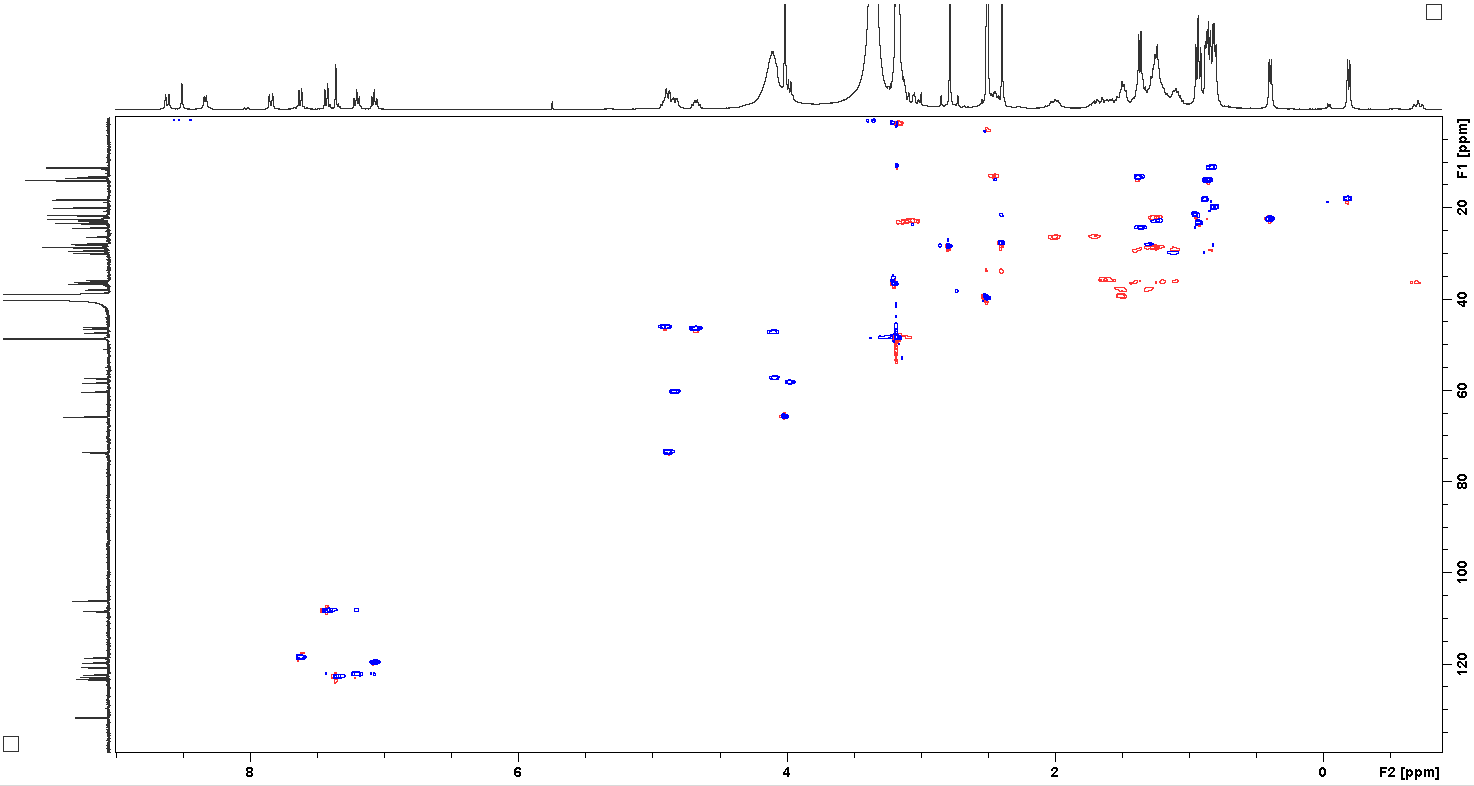


FIGURE S20. HSQC spectrum (DMSO-*d_6_*) of pestahivin C (**3**).


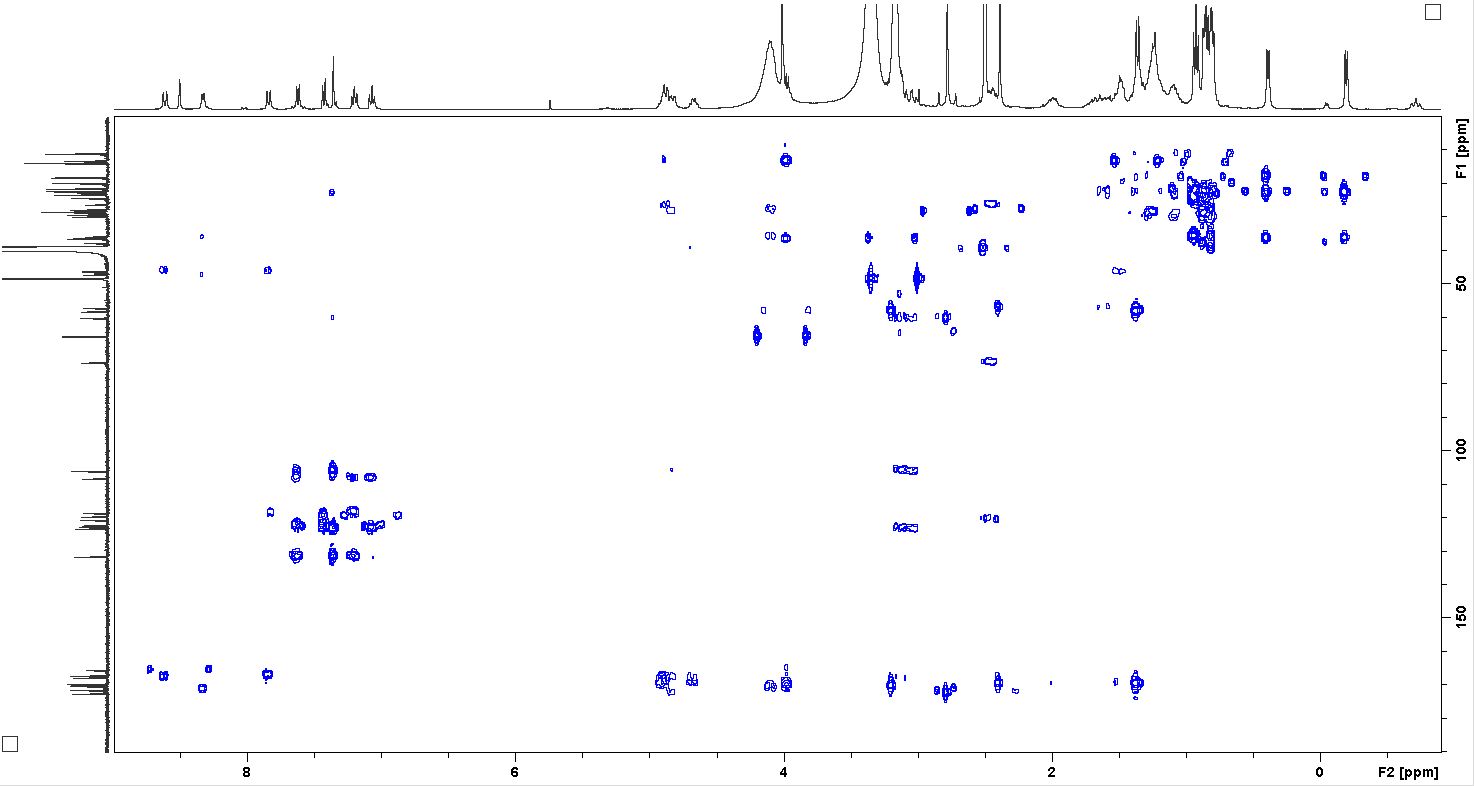


FIGURE S21. HMBC spectrum (DMSO-*d_6_*) of pestahivin C (**3**).


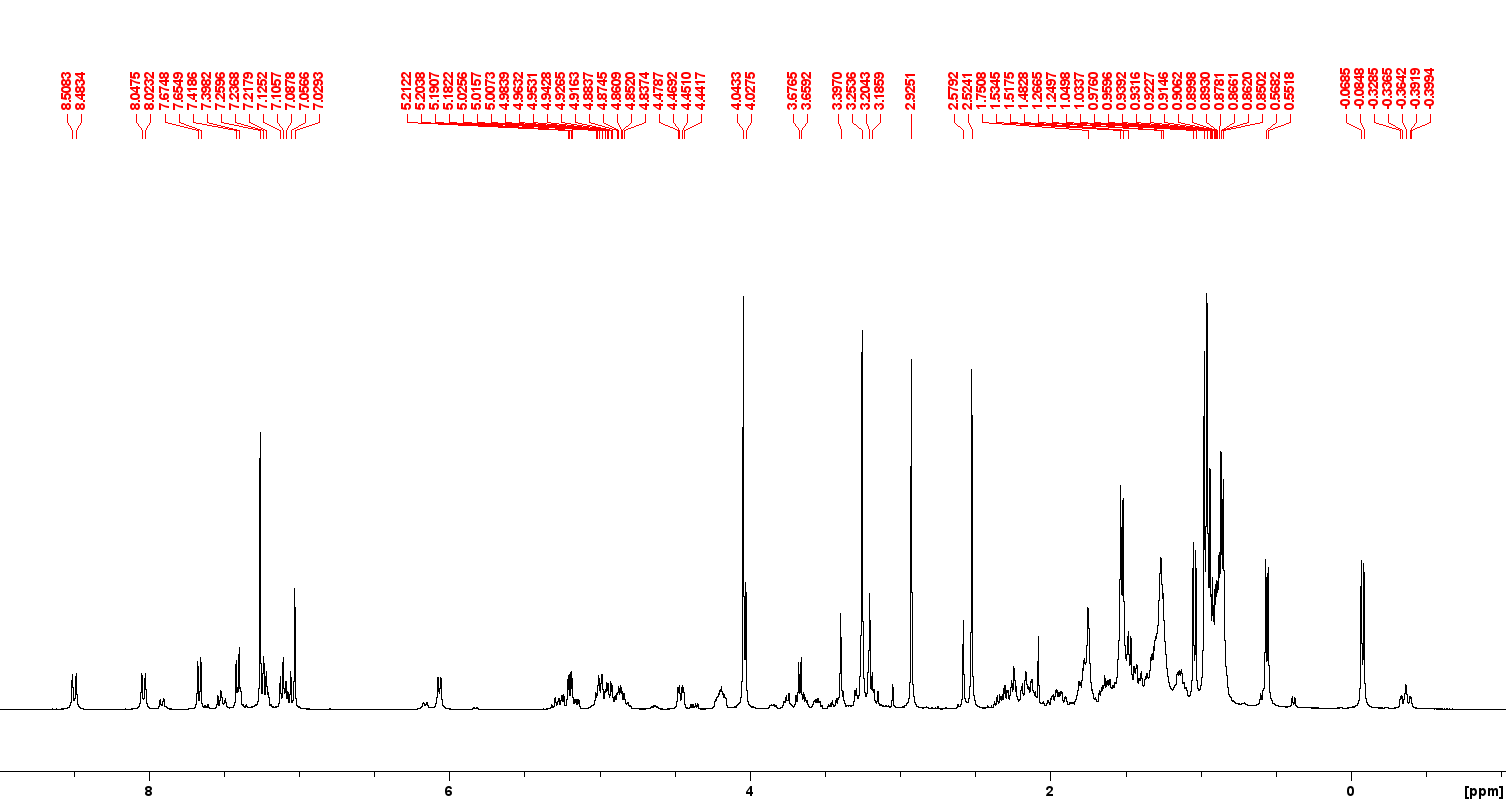


FIGURE S22. ^1^H NMR spectrum (CDCl_3_, 400 MHz) of pestahivin B (**2**).


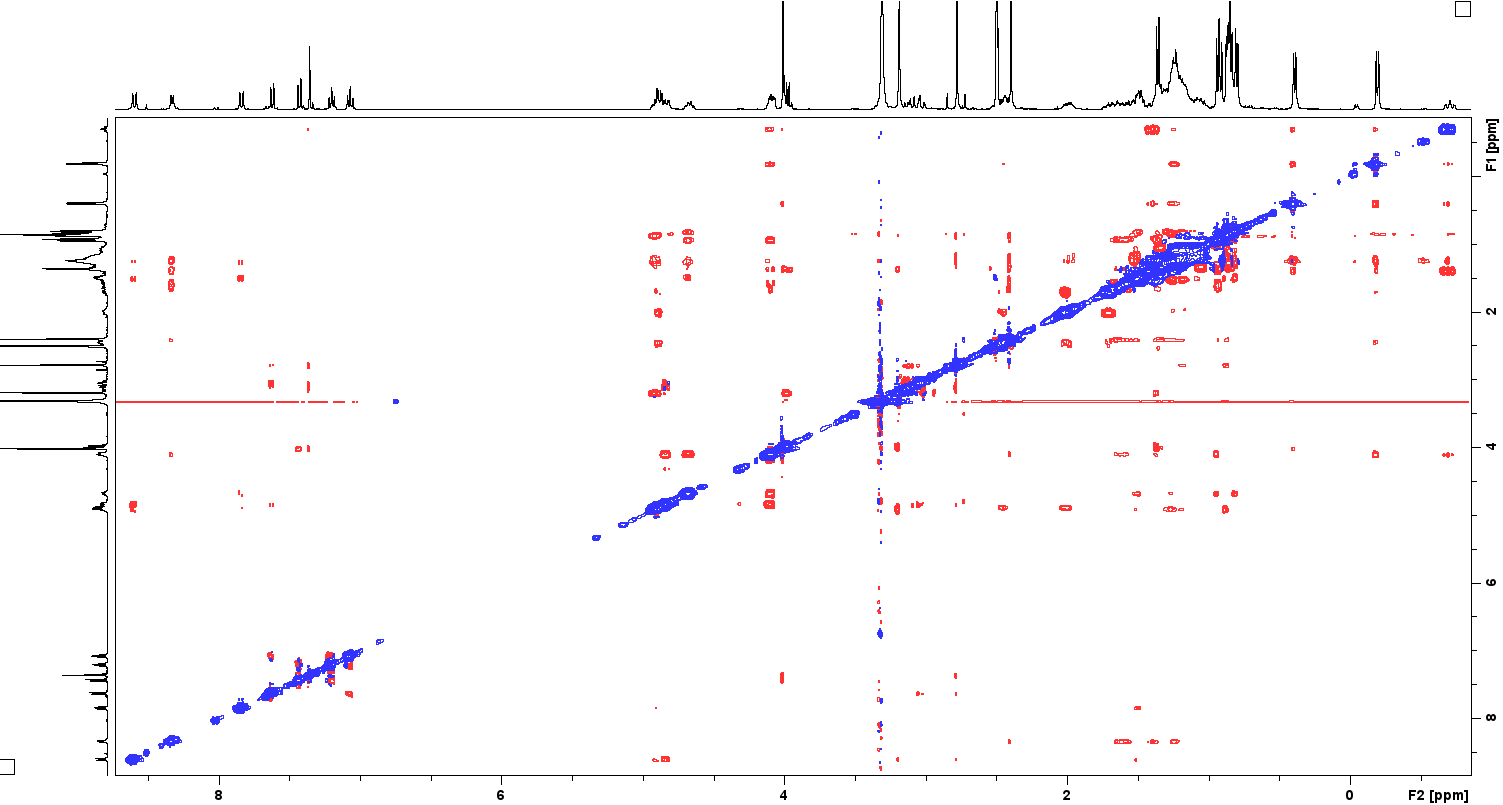


FIGURE S23. ROESY spectrum (DMSO-*d_6_*) of pestahivin (**1**).


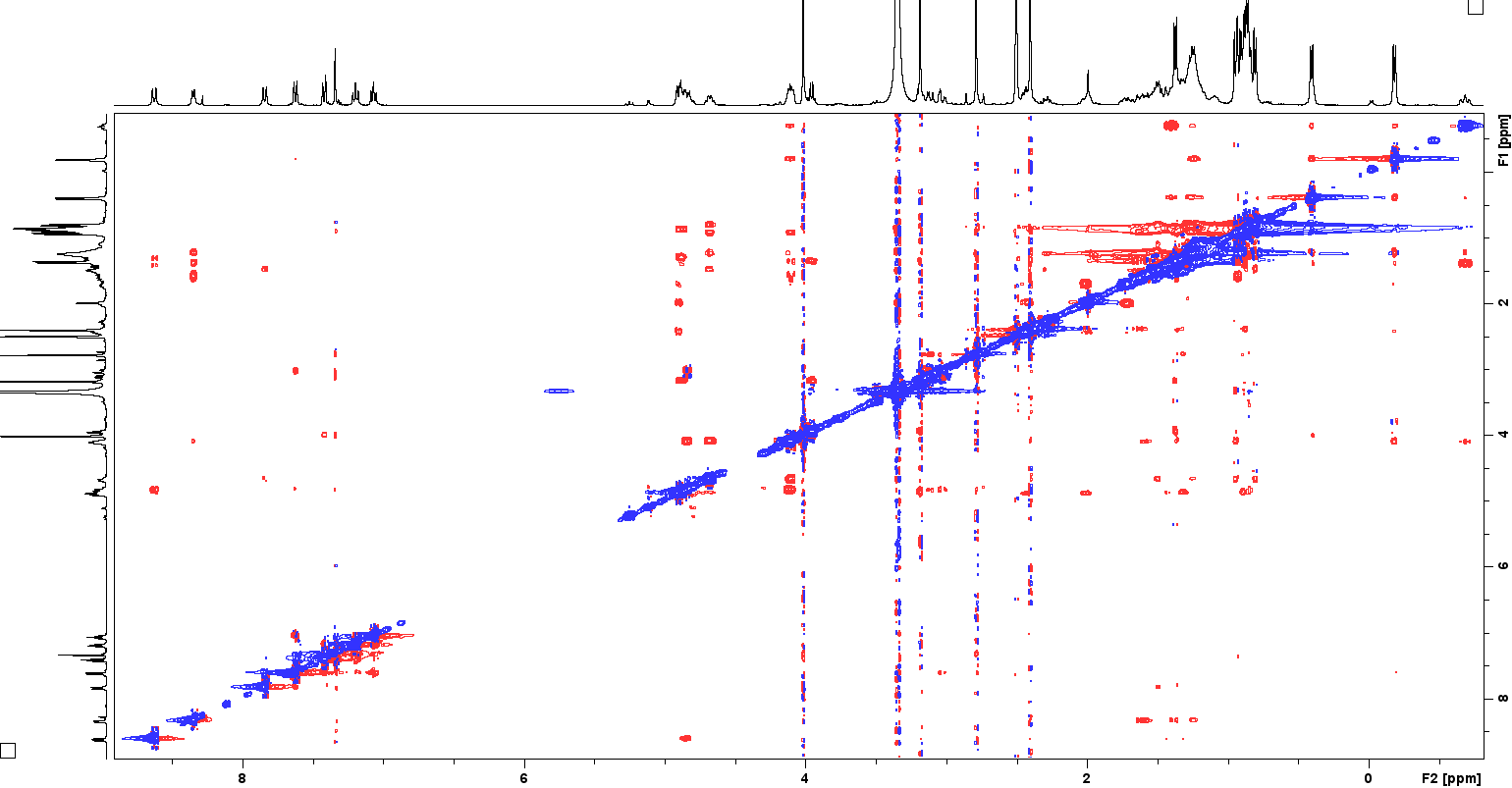


FIGURE S24**.** ROESY spectrum (DMSO-*d_6_*) of pestahivin B (**2**).


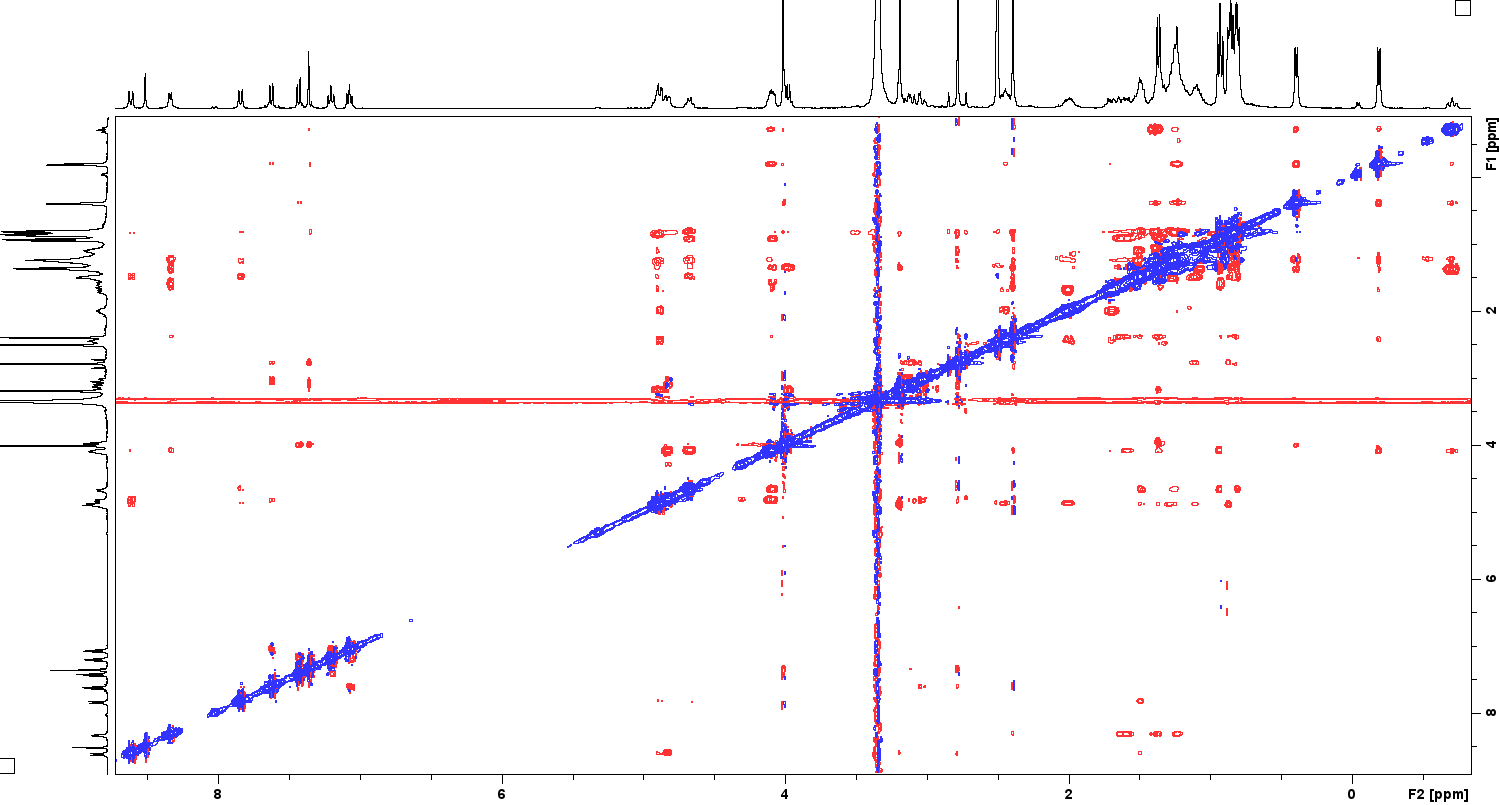


FIGURE S25. ROESY spectrum (DMSO-*d_6_*) of pestahivin C (**3**).


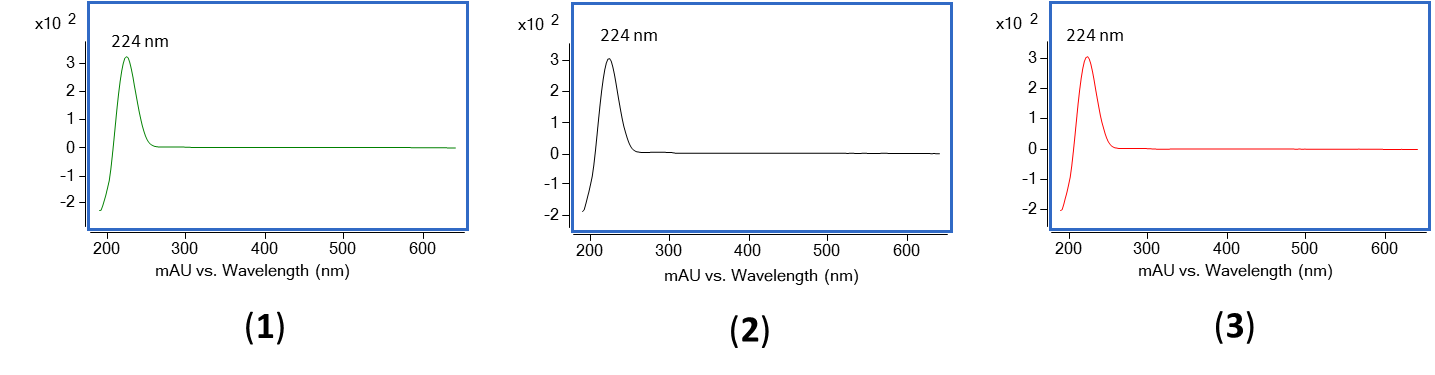


FIGURE S26. UV spectra of compounds **1**–**3**.

FIGURE S27. MS/MS GNPS networking cluster showing molecular correlations between the pestahivins. Data can be seen at <https://gnps.ucsd.edu/ProteoSAFe/status.jsp?task=1d719323a6314d2c87a08ee2cb4b3451>.

FIGURE S28. Dose-response curves for pestahivin (**1**), pestahivin B (**2**) and pestahivin C (**3**) againstmicrobial pathogens: CA10231 = *C. albicans*, AB16404 = *A. brasiliensis,* SA25923 = *S. aureus*and cancer cell lines -A549, MIA PaCa-2, and PANC-1.

TABLE S1. List of primers used in this study.

| **Target** | **Primer** | **Sequence (5' 🡪 3')** | **Observed  product sizes (bp)** | **Reference** |
| --- | --- | --- | --- | --- |
| **Default barcode gene** |  |  |  |  |
| Internal Transcribed Spacer 2 (ITS2) | ITS86F | GTGAATCATCGAATCTTTGAA | ~300 | White et al. (1990); Turenne et al. (1999) |
|  | ITS4 | TCCTCCGCTTATTGATATGC |  | White et al. (1990) |
|  | ITS5 | GGAAGTAAAAGTCGTAACAAGG |  | White et al. (1990) |
| D1D2 region of nr-LSU | LR5 | TCCTGAGGGAAACTTCG | ~950 | Vilgalys and Hester (1990) |
|  | LROR | ACCCGCTGAACTTAAGC |  | Vilgalys and Hester (1990) |
| **Protein-coding gene fragments** |  |  |  |  |
| Translation elongation factor 1-α (TEF-1-α) | EF1-983F | GCYCCYGGHCAYCGTGAYTTYAT | ~700 | Rehner and Buckley (2005) |
|  | EF1-1567R | ACHGTRCCRATACCACCRATCTT |  | Rehner and Buckley (2005) |
| *β*-Tubulin (TUB) | Bt2a | GGTAACCAAATCGGTGCTGCTTTC |  | Glass and Donaldson, 1995 |
|  | Bt2b | ACCCTCAGTGTAGTGACCCTTGGC |  | Glass and Donaldson, 1995 |
| RNA polymerase II second largest subunit (RPB2) | 5F | GA(T/C)GA(T/C)(A/C)G(A/T)GATCA(T/C)TT(T/C)GG |  | Liu et al. 1999 |
|  | 7CR | CCCAT(A/G)GCTTG(T/C)TT(A/G)CCCAT |  | Liu et al. 1999 |

TABLE S2. Strains and NCBI GenBank accession numbers used in this study.

| **Strain** | **Organism** | **Accession numbers** | |
| --- | --- | --- | --- |
|  |  | **ITS2** | **28S rRNA** |
| F6342 | *Colletotrichum cobbittiense* | OP001746 | - |
| F6343 | *Colletotrichum cobbittiense* | OP001747 | - |
| F6344 | *Colletotrichum cobbittiense* | OP001748 | - |
| F6345 | *Colletotrichum cobbittiense* | OP001749 | - |
| F6346 | *Diaporthe sp.* | OP001750 | OP001988 |
| F6348 | *Diplodia cajani* | OP001751 | - |
| F6351 | *Neodevriesia sp.* | OP001752 | OP001989 |
| F6355 | *Colletotrichum cobbittiense* | OP001753 | - |
| F6357 | *Phyllosticta fallopiae* | OP001754 | OP001990 |
| F6369 | *Colletotrichum cobbittiense* | OP001755 | - |
| F6371 | *Colletotrichum cobbittiense* | OP001756 | - |
| F6373 | *Diplodia cajani* | OP001757 | - |
| F6376 | *Phyllosticta fallopiae* | OP001758 | OP001991 |
| F6378 | *Phyllosticta fallopiae* | OP001759 | OP001992 |
| F6379 | *Diaporthe sp.* | OP001760 | OP001993 |
| F6381 | *Colletotrichum cobbittiense* | OP001761 | - |
| F6383 | *Colletotrichum cobbittiense* | OP001762 | - |
| F6384 | *Colletotrichum cobbittiense* | OP001763 | - |
| F6385 | *Colletotrichum cobbittiense* | OP001764 | - |
| F6386 | *Colletotrichum cobbittiense* | OP001765 | - |
| F6387 | *Diplodia cajani* | OP001766 | - |
| F6388 | *Diaporthe sp.* | OP001767 | OP001994 |
| F6389 | *Diaporthe sp.* | OP001768 | OP001995 |
| F6390 | *Colletotrichum cobbittiense* | OP001769 | - |
| F6394 | *Colletotrichum cobbittiense* | OP001770 | - |
| F6406 | *Trichoderma breve* | OP001771 | - |
| F6417 | *Stagonosporopsis sp.* | OP001772 | OP001996 |
| F6419 | *Diaporthe sp.* | OP001773 | OP001997 |
| F6420 | *Colletotrichum cobbittiense* | OP001774 | - |
| F6422 | *Diaporthe sp.* | OP001775 | OP001998 |
| F6423 | *Phyllosticta fallopiae* | OP001776 | OP001999 |
| F6424 | *Colletotrichum cobbittiense* | OP001777 | - |
| F6426 | *Diplodia cajani* | OP001778 | - |
| F6427 | *Leptosillia sp.* | OP001779 | OP002000 |
| F6428 | *Phyllosticta sp.* | OP001780 | OP002003 |
| F6430 | *Aspergillus fumigatus* | OP001781 | OP002001 |
| F6432 | *Colletotrichum cobbittiense* | OP001782 | - |
| F6507 | *Zasmidium sp.* | OP001783 | OP002004 |
| F6509 | *Massaria sp.* | OP001784 | - |
| F6529 | *Corynespora sp.* | OP001785 | OP002002 |
| F9446 | *Preussia sp.* | OP001786 | OP002022 |
| F9447 | *Bartalinia pondoensis* | OP001787 | OP002023 |
| F9448 | *Neopyrenochaeta sp.* | OP001788 | - |
| F9449 | *Robillarda terrae* | OP001789 | - |
| F9452 | *Fusarium hainanense* | OP001790 | - |
| F9456 | *Preussia sp.* | OP001791 | OP002005 |

TABLE S3.

Chemical dereplication results for extracts from two strains F6430 and F9447. Compounds putatively identified by Dictionary of Natural Products database.

| **Strain** | **media** | **Chemical elicitor** | **Target pathogens/ cancer cell lines** | **Active fractions** | **LC-MS dereplication (putative compounds)** **m/z detected** |
| --- | --- | --- | --- | --- | --- |
| F6430 | CF18LB | 100 uM SAHA or  50 µM 5-azacytidine | *A. brasiliensis* | F14 and F15 |   m/z 309.2298 m/z 380.1967   (Deacetoxyfumigaclavine C) (Fumitremorgin C) |
| F6430 | CF18LB | 100 µM SAHA | *S. aureus* | F19 (weak) | m/z 939.4487 and m/z 1009.5256 (unknown) No hit in DNP. |
| F9447 | CF02LB | 50 µM 5-azacytidine or  100 µM SAHA | *A. brasiliensis* | F24 - 26 |   m/z 957.5772; m/z 949.6113 (New Pestahivin analogues)  m/z 977.6425 (Pestahivin) |
| F9447 | CF02LB | 50 µM 5-azacytidine | A549, MIA PaCa-2, PANC-1 | F19, F25-26 |   m/z 957.5772; m/z 949.6113 (New Pestahivin analogues)  m/z 977.6425 (Pestahivin) |

TABLE S4. *Sporocadaceae* family members used in the phylogenetic analyses

| ***Sporocadaceae*** | **Strain / Culture** | **LSU** | **ITS** | ***TEF1α*** | ***RPB2*** | ***β*-tubulin** |
| --- | --- | --- | --- | --- | --- | --- |
| *Bartalinia bella* | CBS 464.61 | MH554264.1 | MH554051.1 | MH554486.1 | MH554964.1 | MH554727.1 |
| *Bartalinia bischofiae* | HKUCC 6534 | AF382367.1 | - | - | - | - |
| *Bartalinia kevinhydei* | MFLUCC 12-0384A | MT477059 | MT477057 | - | - | - |
| *Bartalinia kevinhydei* | MFLUCC 12-0384B | MT477060 | MT477058 | - | - | - |
| *Bartalinia kunmingensis* | KUMCC 18-0178 | MK353085 | MK353083 | - | - | - |
| *Bartalinia lateripes* | HKUCC 6654 | AF382368 | - | - | - | - |
| *Bartalinia laurina* | HKUCC 6537 | AF382369.1 | AF405302.1 | - | - | - |
| *Bartalinia pini* | CBS 143891 | MH554330 | MH554125 | MH554559.1 | MH555033.1 | MH554797.1 |
| *Bartalinia pini* | CBS 144141 | MH554364.1 | MH554170.1 | MH554605.1 | MH555067.1 | MH554843.1 |
| *Bartalinia pondoensis* | CMW 31067 | GU291796 | NR_153599.1 | - | - | - |
| *Bartalinia pondoensis* | CBS_125525 | NG_069931.1 | MH863602.1 | MH554421.1 | MH554904.1 | MH554663.1 |
| *Bartalinia robillardoides* | CBS 122705 | KJ710438.1 | NR_126145.2 | LT853202.1 | LT853152.1 | LT853252.1 |
| *Bartalinia robillardoides* | CBS 122615 | MH554207.1 | MH553989.1 | MH554415.1 | MH554897.1 | MH554657.1 |
| *Bartalinia rosicola* | MFLUCC 17-0645 | MG828988.1 | MG828872.1 | - | - | - |
| *Broomella rosae* | MFLU 16-0244 | MG828990.1 | NR_171819.1 | - | - | - |
| *Broomella vitalbae* | MFLUCC 13-0798 | NG_058681.1 | NR_153610.1 | - | - | - |
| *Discosia artocreas* | CBS 124848 | NG_066214.1 | NR_161081.1 | MH554420.1 | MH554903.1 | MH554662.1 |
| *Heterotruncatella diversa* | CPC 29040 | NG_069589.1 | NR_161107.1 | MH554595.1 | MH555059.1 | MH554833.1 |
| *Heterotruncatella grevilleae* | CPC 16997 | NG_069586.1 | NR_161095.1 | MH554522.1 | MH555002.1 | MH554761.1 |
| *Heterotruncatella spadicea** | CBS 118148 | DQ278928.1 | DQ278913.1 | MH554406.1 | MH554888.1 | MH554648.1 |
| *Hyalotiella spartii* | MFLUCC 13-0397 | NG_059561.1 | NR_137972.1 | KP757764.1 | - | - |
| *Hyalotiella transvalensis* | CBS 303.65 | MH870215.1 | NR_161113.1 | MH554464.1 | MH554942.1 | MH554706.1 |
| *Hymenopleella hippophaeicola* | CBS 140410 | NG_064296.1 | NR_154078.1 | MH554436.1 | MH554919.1 | MH554678.1 |
| *Hymenopleella polyseptata* | CPC 21944 | NG_066223.1 | NR_161100.1 | MH554550.1 | MH555024.1 | MH554789.1 |
| *Morinia acaciae* | CBS 137994 | NG_066162.1 | MH554002.1 | MH554431.1 | MH554914.1 | MH554673.1 |
| *Morinia crini* | CPC 21978 | NG_066224.1 | NR_161101.1 | MH554552.1 | MH555026.1 | MH554791.1 |
| *Neopestalotiopsis rosae* | CBS 101057 | NG_069221.1 | NR_145243.1 | KM199523.1 | MH554850.1 | KM199429.1 |
| *Parabartalinia lateralis* | CBS 399.71 | NG_066216.1 | NR_161087.1 | MH554478.1 | MH554954.1 | MH554719.1 |
| *Pestalotiopsis arceuthobii* | CBS 434.65 | NG_058090.1 | NR_147561.1 | KM199516.1 | - | KM199427.1 |
| *Pestalotiopsis malayana* | CBS 102220 | NG_069217.1 | NR_147550.1 | KM199482.1 | - | KM199411.1 |
| *Pseudosarcostroma osyridicola* | CBS 103.76 | NG_066211.1 | NR_161075.1 | MH554372.1 | MH554851.1 | MH554613.1 |
| *Robillarda terrae* | CBS 587.71 | NG_069197.1 | NR_132902.1 | MH554493.1 | MH554971.1 | MH554734.1 |
| *Seimatosporium cornii* | MFLUCC 14-0467 | NG_059570.1 | NR_156597.1 | - | - | - |
| *Seiridium papillatum* | CBS 340.97 | NG_057755.1 | NR_156621.1 | MH554468.1 | LT853150.1 | LT853250.1 |
| *Sporocadus rotundatus* | CBS 616.83 | NG_069584.1 | NR_161091.1 | MH554496.1 | MH554974.1 | MH554737.1 |
| *Strickeria kochii* | CBS 140411 | MH878680.1 | NR_154423.1 | MH554437.1 | MH554920.1 | MH554679.1 |
| *Trochilispora schefflerae* | COAD 2371 | NG_069571.1 | NR_165881.1 | MH231216.1 | - | MH231215.1 |
| *Truncatella angustata* | CPC 21359 | NG_066221.1 | NR_164272.1 | MH554546.1 | MH555021.1 | MH554785.1 |
| *Bartalinia* sp. | F9447 | OP002023 | OP001787 | OP828689 | OP828688 | OP828690 |

**syn: Truncatella hartigii*

**Supplementary references**

Carbone, I., and Kohn, L.M. (1999). A method for designing primer sets for speciation studies in filamentous ascomycetes. *Mycologia* 91, 553-556. doi:10.2307/3761358.

O’Donnell, K., Nirenberg, H.I., Aoki, T., and Cigelnik, E. (2000). A Multigene phylogeny of the *Gibberella fujikuroi* species complex: Detection of additional phylogenetically distinct species. *Mycoscience* 41, 61-78. doi:10.1007/BF02464387.

Felsenstein J. (1985). Confidence limits on phylogenies: an approach using the bootstrap. *Evolution* 39, 783–791. https://doi.org/10.2307/2408678

Glass, N.L., and Donaldson, G.C. (1995). Development of primer sets designed for use with the PCR to amplify conserved genes from filamentous ascomycetes. *Appl. Environ. Microbiol.* 61, 1323-1330. doi:10.1128/aem.61.4.1323-1330.1995.

Kumar, S., Stecher, G. and Tamura, K. (2016). MEGA7: Molecular Evolutionary Genetics Analysis version 7.0 for bigger datasets. Mol. Biol. Evol. 33, 1870-1874. https://doi.org/10.1093/molbev/msw054

Liu, Y. J., Whelen, S., and Hall, B. D. (1999). Phylogenetic relationships among ascomycetes: Evidence from an RNA polymerse II subunit. *Mol. Biol. Evol.* 16, 1799-1808. doi:10.1093/oxfordjournals.molbev.a026092.

Rehner, S,A,, Buckley, E. (2005) A *Beauveria* phylogeny inferred from nuclear ITS and EF1- sequences: evidence for cryptic diversification and links to *Cordyceps teleomorphs*. Mycologia 97, 84–98. doi: 10.3852/mycologia.97.1.84

Perrone G, Stea G, Epifani F, Varga J, Frisvad JC, Samson RA (2011) *Aspergillus niger* contains the cryptic phylogenetic species *A. awamori*. Fungal Biology 115, 1138-1150.  DOI: 10.1016/j.funbio.2011.07.008

Tamura, K., Nei, M. and Kumar, S. (2004). Prospects for inferring very large phylogenies by using the neighbor-joining method. *Proceedings of the National Academy of Sciences*, 101, 11030-11035. https://doi.org/10.1073/pnas.040420610

Turenne, C.Y., Sanche, S.E., Hoban, D.J., et al (1999) Rapid identification of fungi by using the ITS2 genetic region and an automated fluorescent capillary electrophoresis system. J Clin Microbiol 37, 1846–1851

Vilgalys, R., and Hester, M. (1990) Rapid genetic identification and mapping of enzymatically amplified ribosomal DNA from several *Cryptococcus* species. J. Bacteriol. 172, 4238-4246. DOI: 10.1128/jb.172.8.4238-4246.1990

White, T.J., Bruns, T.D., Lee, S.B., and Taylor, J.W. (1990) Amplification and direct sequencing of fungal ribosomal RNA genes for phylogenetics. Pp. 315-322 In: PCR Protocols: A Guide to Methods and Applications, eds. Innis, M. A., D. H. Gelfand, J. J. Sninsky, and T. J. White. Academic Press, Inc., New York.
